# A comparative analysis of urban forests for storm-water management

**DOI:** 10.1038/s41598-023-28629-6

**Published:** 2023-01-26

**Authors:** Mohammad A. Rahman, Yanin Pawijit, Chao Xu, Astrid Moser-Reischl, Hans Pretzsch, Thomas Rötzer, Stephan Pauleit

**Affiliations:** 1 Strategic Landscape Planning and Management, School of Life Sciences, Weihenstephan, Technische Universität München, Emil-Ramann-Str. 6, 85354 Freising, Germany; 2 Research Center for Eco-Environmental Engineering, Dongguan University of Technology, Daxue Road 1, Dongguan, 523808 China; 3 Forest Growth and Yield Science, School of Life Sciences, Weihenstephan, Technische Universität München, Hans-Carl-von-Carlowitz-Platz 2, 85354 Freising, Germany

**Keywords:** Urban ecology, Plant evolution

## Abstract

Large-scale urban growth has modified the hydrological cycle of our cities, causing greater and faster runoff. Urban forests (UF), i.e. the stock of trees and shrubs, can substantially reduce runoff; still, how climate, tree functional types influence rainfall partitioning into uptake and runoff is mostly unknown. We analyzed 92 published studies to investigate: interception (I), transpiration (T), soil infiltration (IR) and the subsequent reduction in runoff. Trees showed the best runoff protection compared to other land uses. Within functional types, conifers provided better protection on an annual scale through higher I and T but broadleaved species provided better IR. Regarding tree traits, leaf area index (LAI) showed a positive influence for both I and T. For every unit of LAI increment, additional 5% rainfall partition through T (3%) and I (2%) can be predicted. Overall, runoff was significantly lower under mixed species stands. Increase of conifer stock to 30% in climate zones with significant winter precipitation and to 20% in areas of no dry season can reduce runoff to an additional 4%. The study presented an overview of UF potential to partition rainfall, which might help to select species and land uses in different climate zones for better storm-water management.

## Introduction

Rapid and large-scale urban growth has modified the hydrological cycle of our cities by replacing natural vegetation surfaces with impervious surfaces, causing greater and faster runoff^1,2^. Barron et al.^3^ reported an increase of the annual runoff coefficient from 1% in pre-development condition to 39% and reduction in evapotranspirative water loss from 443 to 154 mm after urbanization in an urbanized watershed in Western Australia. Between 1980 and 2018, direct economic impacts of urban flooding has exceeded US $1 trillion globally, with the loss of hundreds of thousands of lives^4^. In the context of advancing climate change and increasing storm intensity, cities have therefore been prompted to invest in ways to naturally capture, store and slowly release runoff through "green infrastructure"^5^. In Europe, the concept of nature-based solutions (NBS) is emphasized in policies to provide a wide range of ecosystem services^6^ thus improving urban sustainability and resilience^5,7,8^. In recent years, concepts of NBS for storm-water management have appeared under many terms worldwide, i.e., Sustainable Urban Drainage System, Low Impact Developments in US, Blue-Green Cities in the UK, Water Sensitive Urban Design in Australia^9^, Low Impact Developments Urban Design in New Zealand and Sponge City in China^10^. Despite the differences in the names and objectives, each of the solutions share a common ground, aiming to integrate GI into urban areas to restore natural water cycles.

Vegetation can significantly reduce runoff^11,12^; however, concepts and policies for urban forestry as NBS, peaked only in the mid-1990s^13^. Still, research on the interaction between urban forests, i.e. the urban stock of trees, and storm-water has been relatively understudied compared to other topics such as microclimatic amelioration^14^, air quality and carbon sequestration benefits^15–17^. Urban forests return water to the atmosphere through interception and evaporation, regulate ground-water flow by through-fall (rain that falls through the canopy) and stem flow (water that flows down the trunk or stem) leading towards soil infiltration^18^. Finally transpire water out of the soil, leading to increased soil water‐holding capacities^19^. Arguably, transpiration is the major component of water partitioning at annual scale though that can be different in closed canopy multilayered forests compared to isolated urban trees without any humus layer or rows of hedgerows or grass lawns and higher atmospheric demand^20^.

From an urban hydrological point of view, canopy interception is the most important effect and trees with their greatest stature can provide the greatest benefit in this respect. In forest condition, interception could be as large as 50% that of gross precipitation^21^. Whereas for individual trees this has been variously reported between 15 and 28%^22^. Depending on tree species, their canopies can retain between 0.03 and 2.24 mm H_2_O m^−2^ of leaf area as shown in a rainfall simulator study^23^. The whole process of rainfall partition is affected by three main factors: nature and magnitude of the rainfall event, functional types of vegetation, and weather conditions^22^. In general, conifers intercept and evaporate 20–40% of annual rainfall while deciduous forests intercept 10–20%^24^, compared to 15–32% by mixed forest stands^25^.

Reducing surface-runoff, increasing soil infiltration for further enhancing transpiration (hence cooling effect) cannot be promoted by a one-size-fits-all solution. Runoff from conifers is usually lower compared to deciduous stands, in particular where winter rainfall is significantly higher. Again, considering individual storm events, deciduous stands might retain more water than conifers^26^. Apart from tree crown structure and rainfall intensity and duration, overall water detention is largely dependent on the climatic conditions i.e., radiation, air humidity temperature, and wind speed^18^.

Studies on rainfall partitioning are largely fragmented, focusing on just one or few parts of the partition, with a wide range of values across studies. Moreover, global scientific discourse on urban forestry is limited to few regions. Ostoić and van den Bosch^13^ reported 58% of all publications related to urban forestry is from the USA alone. Thus, comprehensive understanding of the magnitude of surface-runoff reduction potentials of different urban forests is mostly missing. Notably, vegetation with high species and functional diversity can reduce runoff mostly based on the canopy structure attributes. However, belowground traits in terms of improving the soil infiltration potential is mostly ignored. Importantly, the influence zone of a tree stem can be much higher than their canopy extent. Chandler and Chappell^27^ showed significant positive influence on increasing soil infiltration of an old oak (*Quercus robur*) up to 10 m from the main stem. Therefore, knowledge concerning the rainfall partitioning through interception, infiltration, transpiration as part of the annual water balance in an urban context over higher spatial and temporal scales is needed. Such overview would provide storm water managers with a useful insight on different climate regions and different functional types of vegetation.

The objective of this study is to present an overview of how urban forests partition rainfall on an annual basis in different climate regions, to understand the implications of climate and different functional types of greenspaces on storm-water management. The research questions are:

1. How does climate affect rainfall partitioning into uptake and runoff of the urban forest?
2. How can different functional types of urban forests influence the reduction in storm-water runoff?
3. How do characteristics of tree species affect storm-water partitioning?

## Methods

### Search and selection of studies

Relevant studies were searched using internet search engines and websites of environmental organizations. Due to the lack of data in urban settings, forest stands were also included to fill the data gaps. Moreover, runoff coefficient and infiltration potential are mostly measured at landscape level, information on tree species were difficult to isolate. Consequently, runoff coefficient and soil hydraulic conductivity values were collected from studies covering different land use and land cover i.e., mixed forest, monospecific plantation, afforestation (1–30 years), and open spaces including bare soil in various climate zones. Keywords used were urban OR trees OR forest OR plantation OR storm-water OR interception OR infiltration OR evapotranspiration OR transpiration OR runoff OR infiltration rate OR hydraulic conductivity. Both empirical and modelling studies published between 1995 and 2021 were considered. The selection criteria following Stewart^28^ includes: (1) studies regarding specific trees or shrubs in or around cities with quantifiable data on interception, evapotranspiration, infiltration or runoff; (2) specific land use containing values that can be converted into percentage of the total precipitation. Through-fall and stem flow were not explored since both end up in the soil and contribute either to runoff or infiltration.

Finally, 92 studies were selected for in-depth analyses (S-Table 1); 28 studies (81 data points) were considered for interception (50% from urban settings), 19 studies (67 data points) were selected for transpiration (75% from urban settings). There were 36 studies (201) data points for runoff coefficient and 9 studies (42 data points) for infiltration.

### Classification

#### Climate

**Figure 1:**
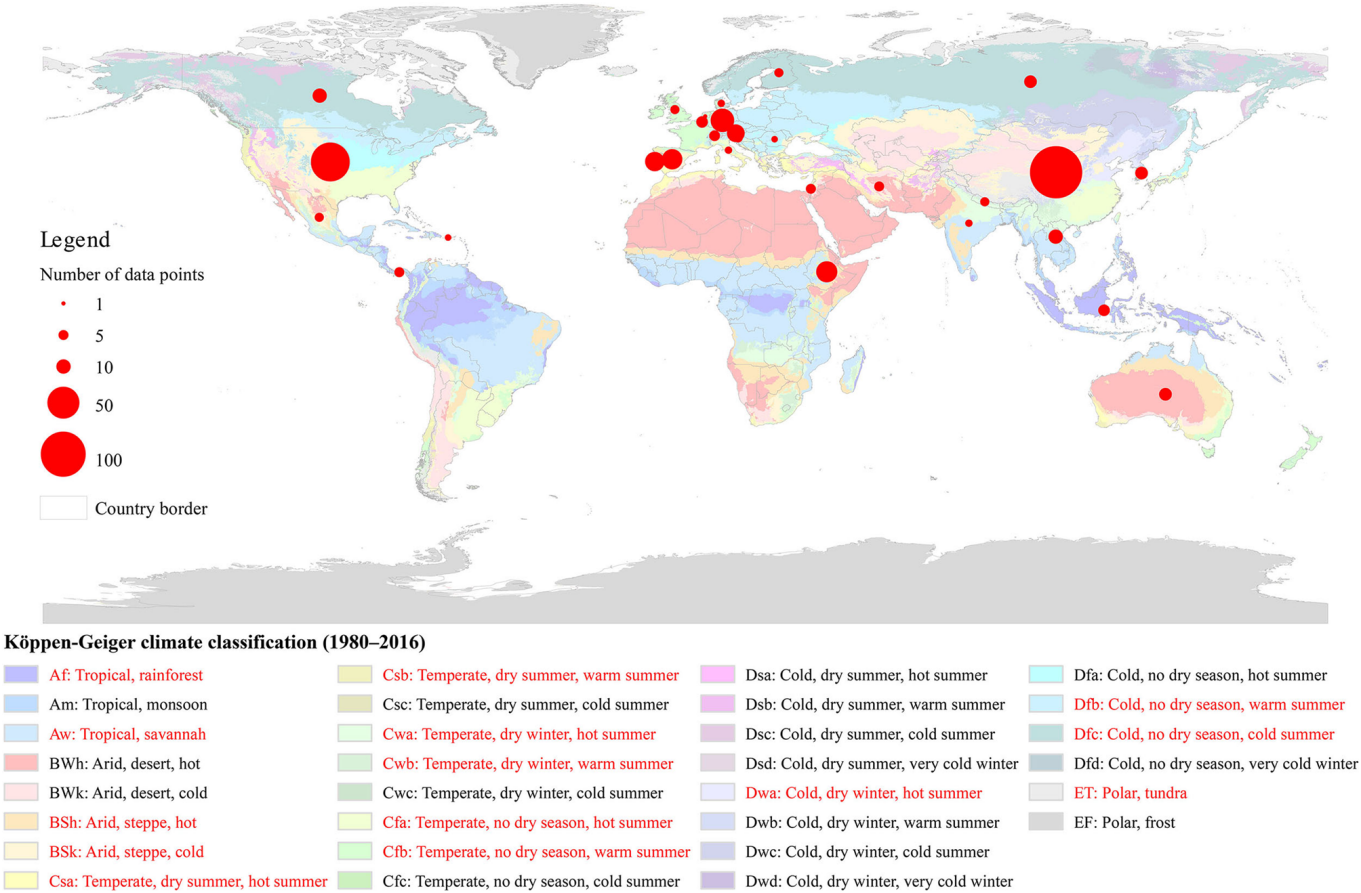
Overview of the Köppen–Geiger climate classes including the considered climate zones in this study (red colored). The sizes of the circles are proportional to the number of data points collected from each country.

Location of each data point was assigned to a climate classification based on Beck et al.^29^’s updated Köppen–Geiger climate classification (Fig. 1). For analysis, only the first two letters (climate group and seasonal precipitation) for climate groups C and D were used. Data from climate groups A, B and E were classified into three broad climate groups, without considering subgroups, as there were less data points. For studies that did not report climate classification, the place was located through Google Maps and the approximate location was compared manually.

#### Tree and land use

Trees were categorized into five groups: broadleaved evergreen (BE); broadleaved deciduous (BD); evergreen coniferous (EC), deciduous conifers (DC) and shrubs (S). For interception, along with the volume, rainfall duration and intensity were also considered.

Land use under infiltration and runoff were categorized into 15 groups of stands/types: mixed forest (MiF); afforested land (Af); mixed coniferous (MiC); mixed deciduous (MiD); mono fir (MoF); mono spruce (MoS); mono pine (MoP); mono beech MoB); mono oak (MoO); mono broadleaved (MoBr: in the case of a mix of several species, but limited to broadleaved); mono Eucalyptus (MoE); agricultural land (Ag); shrubland (Sh); grassland (Gr) and bare soil (Bs) (S-Table 1). To understand the broad differences between broadleaf and conifers, several land uses were grouped together under these two broad categories. ‘Broadleaf’ group includes: MiD; MoO; MoBr and MoE. ‘Conifers’ group includes: MiC; MoF; MoS and MoP.

### Normalization

Transpiration rates in mm day^−1^ was analyzed either as per canopy or plot area. In case of missing values, sapwood area (water conducting xylem tissues within the main stem), diameter at breast height and canopy or plot area were used. Where only flux density was provided, transpiration rate (Ec) was calculated (Eq. 1) following Rahman et al.^6,14^:

1 $${E}_{c}=\frac{{J}_{s} \times {A}_{s} }{1000}\times {A}_{c}$$

where Js is the daily sap flux density in g H_2_O m^−2^ s^−1^, A_s_ is the sapwood area in cm^2^, and A_c_ is the canopy area in m^2^.

For infiltration, saturated hydraulic conductivity (Ks) values in mm h^−1^ were used. In cases of several values of tree types or land uses i.e., interception rate for different seasons (leafed/leafless period) or runoff coefficient for different rainfall intensities, the mean value was used to represent the annual rate. When independent variables such as rainfall intensity or leaf area index (LAI) were provided without a corresponding dependent variable, the mean value was used to represent the dataset.

Normalization was applied to runoff coefficient values to adjust the effects of plot size and rainfall intensity. Plot size normalization values were based on Moreno-de las Heras et al.^30^, who conducted runoff experiments on plot sizes with 1, 2, 3 and 15 m (micro-catchment) lengths on five different slopes, three of which were degraded and two were less-degraded. The runoff coefficient values from the results were summed within the same plot length categories and a percentage reduction was calculated (S Table 2). Consequently, only runoff coefficient for plot sizes < 1–3 m^2^ were normalized to the 15 m length equivalent following Moreno-de las Heras et al.^30^ (S-Table 2). Plot lengths between 4 and 15 m were not considered. Plot sizes with lengths over 15 m were not normalized. Runoff coefficient values of degraded slopes were applied to bare soils, grassland and shrub land, whereas values of less-degraded slopes to other land uses with plant cover. The normalized runoff coefficient were calculated (Eq. 2):

2 $$Rn=Ro-(Ro\times r)$$

where Rn is the normalized runoff coefficient, R_o_ is the runoff coefficient and *r* is the percentage reduction as in S-Table 2.

Rainfall intensity over 45 mm h^−1^ was normalized following Wu et al.^31^ (S-Table 3) (Eq. 3):

3 $${R}_{n}=\frac{{R}_{o}}{r}$$

where Rn is the normalized runoff coefficient, R_o_ is the runoff coefficient and *r* is the increased rate of runoff coefficient from one rainfall intensity to the other.

Slope effects were not normalized since the results of many studies were inconclusive^31^.

### Analysis

The water balance of the rainfall partitioning was estimated as *Pg* = *I* + *T* + *R* + *IR*, where Pg is the annual precipitation as 100%, I is the annual interception rate as a ratio to the precipitation (%), T is the annual transpiration rate per canopy area as a ratio of the annual precipitation (%), R is the runoff coefficient (%) and IR is the infiltration rate which cannot be expressed as a ratio to precipitation; therefore, IR was obtained as *IR* = *Pg* − (*I* + *T* + *R*).

All the 36 studies (S-Table 1) considered for runoff coefficient used empirical data. Similarly, all the 19 studies considered for transpiration (S-Table 1) used field measured transpiration rates either using leaf porometer, stem flux density or eddy covariance data. For annual precipitation data, the studies used 30 years average annual precipitation from a meteorological station (some studies did not explicitly mention about 30-year average and meteorological stations name). Since transpiration data was mostly collected during the foliage time of the year, we paid attention to the data collection period. All the transpiration data within the investigated studies covered the rainy seasons (higher soil moisture potential), hence, the chance of underestimation is low.

Out of 28 studies (S-Table 1) considered for interception, ten used different modelling approaches. Among different models, the Rutter^32^ and Gash models^33^ are most commonly used for rainfall interception studies. Véliz-Chávez et al.^34^ compared these two models, validated the model results with the measured data and reported that the Gash model is more accurate. Among nine modelling studies, six studies namely: Fan et al.^35^; Ghimire et al.^36^; Pereira et al.^37^; Pypker et al.^38^; Ringgaard et al.^39^; Price and Carlyle-Moses^40^ used a revised Gash model and validated the simulations with measured data. Considering the limitations of the Rutter and Gash models to predict interception and water retention in single trees, Guevara-Escobar et al.^22^ used semiariograms as descriptors of through-fall variability for modeling water flux in a surface response. Some studies used liner regression models (e.g., Fathizadeh et al.^41^) to estimate canopy storage from the relationship between cumulative gross precipitation and through-fall. Livesley et al.^42^ used regression analyses between gross rainfall and through-fall and stem flow to calculate interception. Similarly, Xiao and McPherson^43^ used a single tree model considering the tree morphological (dimension, leaf surface area) and meteorological data as input to predict interception by different tree species at different stages.

In terms of rainfall amount, 9 out of 28 studies had no cited sources or data were averages from less than 10 years. 13 out of 28 studies have more than 10-years averages, at least 18 years. The remaining studies have cited meteorological data whereby the number of years was not explicitly specified. However, the percentage of interception were all calculated based on the incident precipitation, not on the average annual rainfall. All the studies used measured data except Xiao and McPherson^43^, where the authors used computer simulations using a 25-year rainfall event. For studies with measurement periods of less than 12 months, interception rate was taken either as mentioned in the paper or was calculated relative to the specific months measured.

Data extraction was done using WebPlotDigitizer^44^ and data analysis using RStudio (1.3.1073)^45^ with packages read_xl; tidyverse; aov; ggplot, cowplot, gpubr and dplyr.

Data were first assessed for normal distribution, and there was no violation of variance homogeneity and later subjected to one or two way ANOVA. To understand the relationship between morphological characteristics or soil physical properties liner regression analyses were carried out. Following García-Palacios et al.^46^ and Le Provost et al.^47^, the relative importance (effect) of the influencing factors based on a multivariate regression model was evaluated. Means were reported significant when p < 0.05.

## Results

### Interception

**Figure 2:**
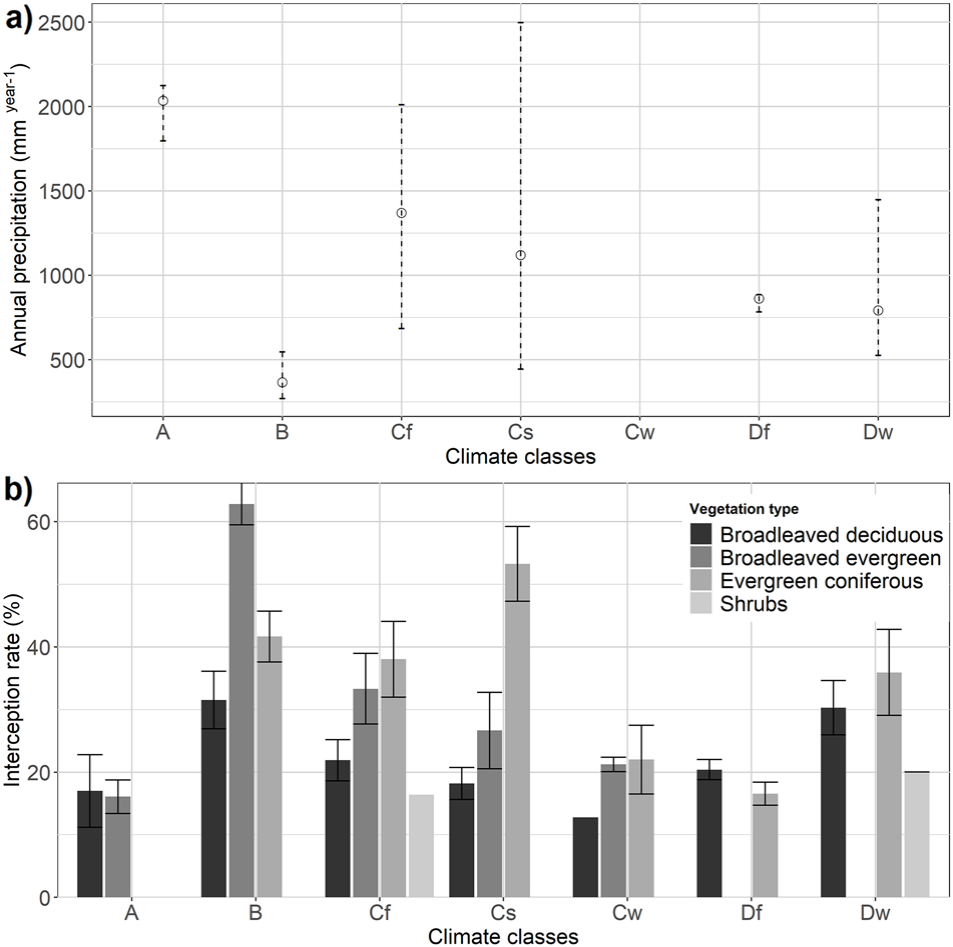
(**a**) Range and mean (circle) annual precipitation of different climate zones used in this review. (**b**) Bar chart showing the interception rate of different vegetation types at different climate zones (± SE). Here, A stands for tropical, B for arid, Cf temperate with no dry season, Cs temperate with dry summer, Cw temperate with dry winter, Df continental with no dry season, Dw continental with dry summer and E for polar climate.

Annual rainfall amounts showed a strong influence on the interception rate, the lower the rainfall, the higher the values for the interception rate, as for example in arid climate (B). An exception was the climate zone DF (Ontario, Canada). Overall, the interception rate was significantly higher for evergreen coniferous (mean, µ = 39%), followed by broadleaved evergreen (µ = 28%), broadleaved deciduous (µ = 23%) and shrubs (µ = 19%) (p = 7.6e^−04^) (Fig. 2). Only in arid climate broadleaved evergreen showed a significantly higher interception rate (µ = 63%) compared to evergreen conifers (µ = 42%). Both climate and vegetation types showed separate (p = 0.001 for climate and p = 6.72e^−05^ for vegetation type) and interaction effect (p = 0.02) on interception rate.

### Transpiration

**Figure 3:**
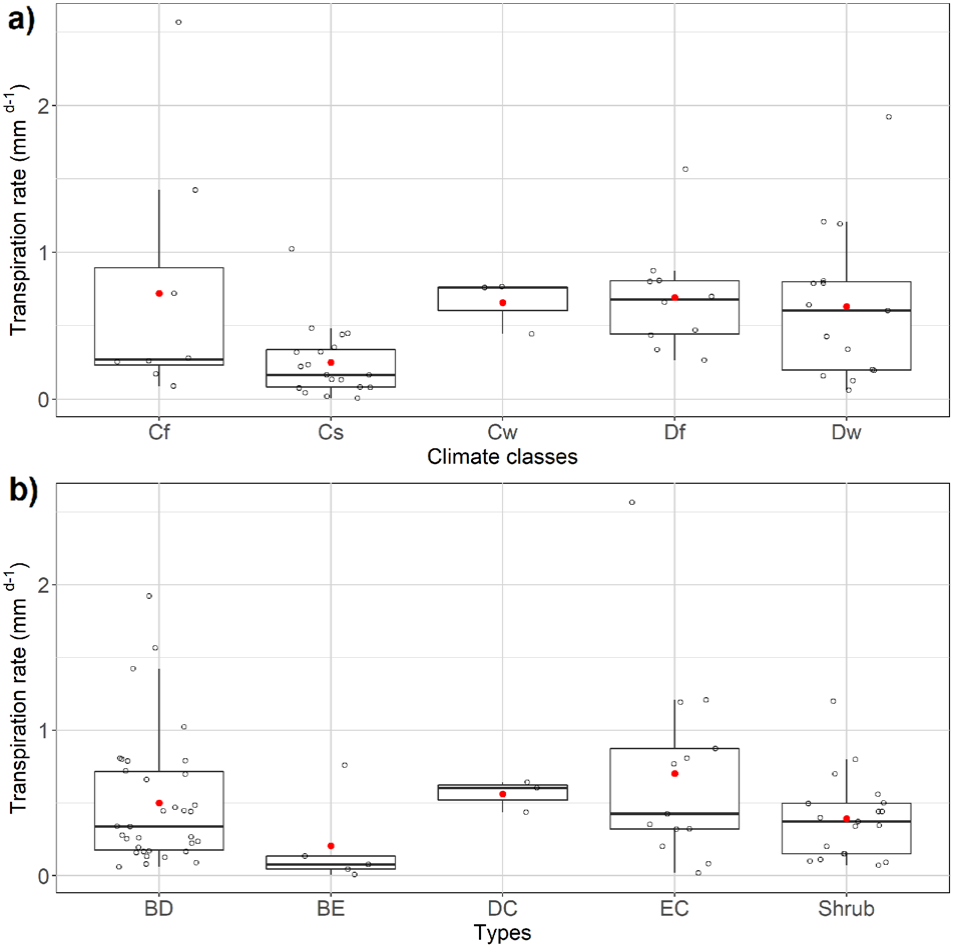
Transpiration rate on an annual scale (**a**) by climate zones; (**b**) by tree types (empty dots show the distribution of individual data points, red dot = mean, dark line = median).

Due to the lack of transpiration data in particular across climate zones, only climate zones C and D were compared. The range of transpiration rate varied between 0.1 and 2 mm day^−1^ at annual scale. The highest mean transpiration was found in Df region (0.7 mm day^−1^) and the lowest in Cs (0.3 mm day^−1^) (Fig. 3a). In general, coniferous evergreen trees showed the highest mean transpiration rate (0.7 mm day^−1^) compared to broadleaved deciduous trees (0.5 mm day^−1^), shrubs (0.4 mm day^−1^) and broadleaved evergreen (0.2 mm day^−1^) (Fig. 3b).

### Morphological traits influencing interception and transpiration

**Figure 4:**
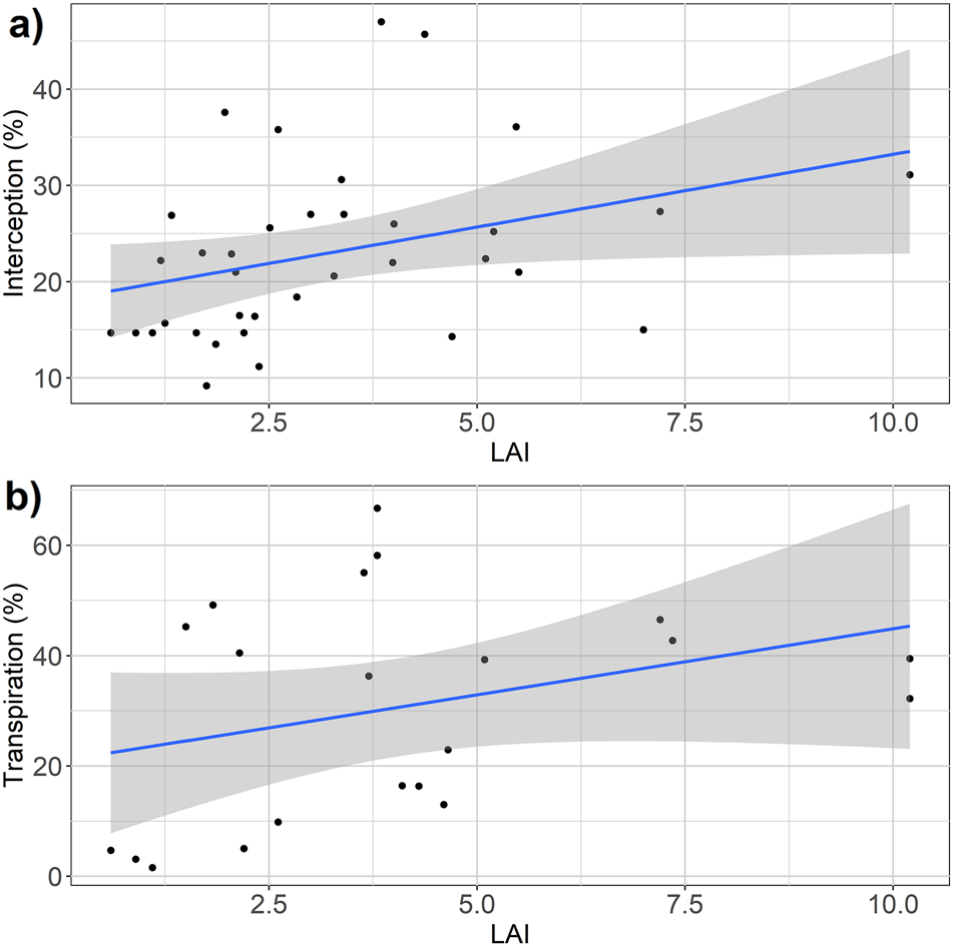
Relationship between leaf area index (LAI) for trees with (**a**) interception rate; (**b**) transpiration rate.

Both interception and transpiration rates were significantly related to DBH (p = 0.03, n = 35 for interception and p = 0.01, n = 53 for transpiration), though results were inconsistent. The significance level varied between climate zones, vegetation functional types and between rural and urban sites. LAI showed a consistent pattern over all independent variables (Fig. 4).

### Infiltration, runoff and water balance

**Figure 5:**
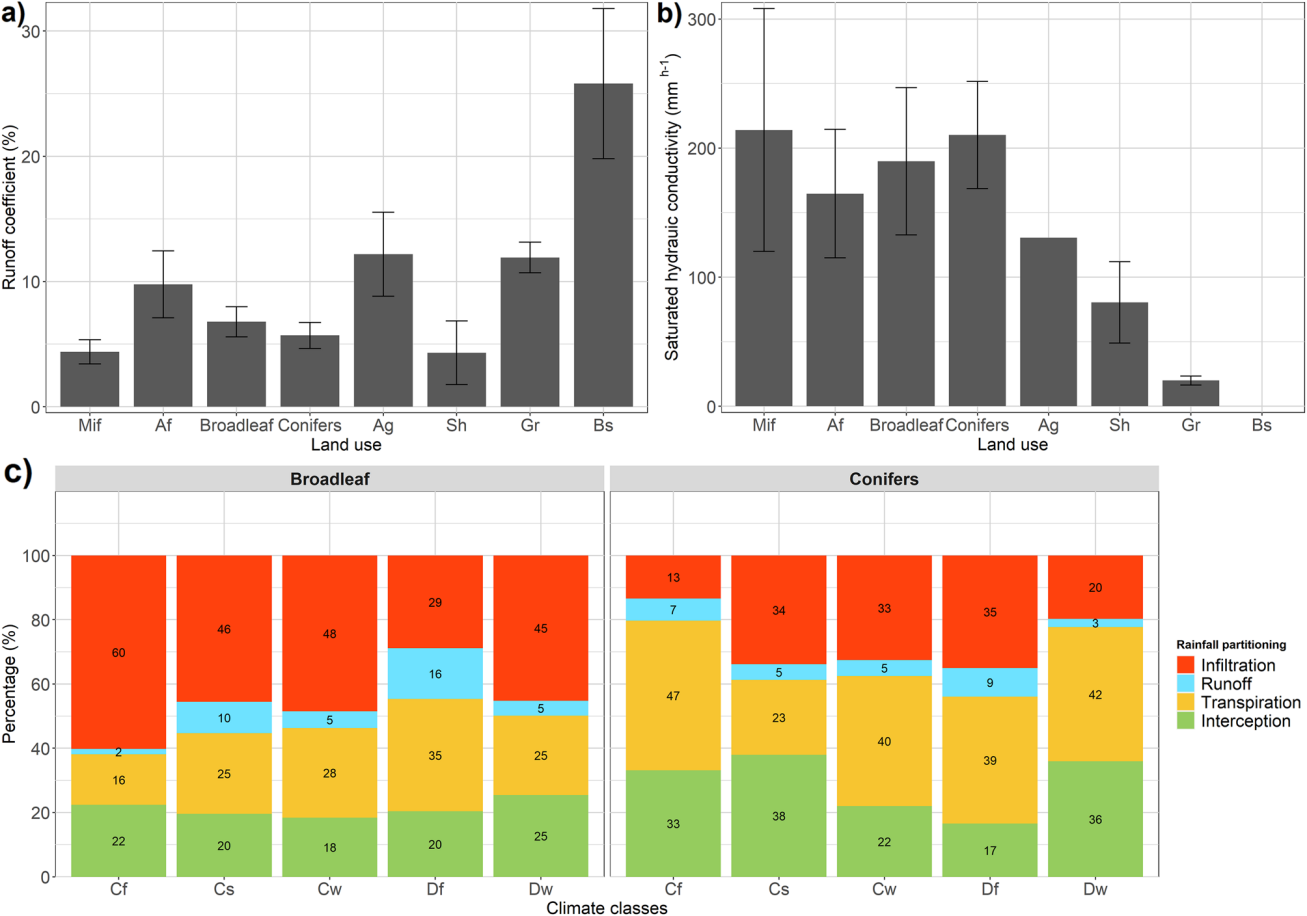
Runoff coefficient (**a**) and saturated hydraulic conductivity (**b**) by land use (*Mif* mixed forest, *Af* afforestation, *broadleaf* mix broadleaved stands, *conifers* mix coniferous stands, *Ag* agriculture, *Sh* shrubland, *Gr* grassland, *Bs* bare soil) and water balance (**c**) as percentage of rainfall by climate zones for broadleaf trees and conifers.

Overall, forests showed substantially higher Ks compared to agriculture, shrubs and grasslands (Fig. 5b). The highest Ks was found in broadleaved stands followed by mixed and coniferous stands and least for afforested stands (Fig. 5b). Mixed forest, mixed deciduous, spruce, pine and oak stand had the highest Ks at 0–10 cm soil depth, whereas Eucalyptus stand had the highest Ks at 40 cm. Shrub and grassland both indicated negligible Ks over 20–30 cm depth. Bulk density were negatively related to porosity (p = 2.2e^−16^) and in turn, both bulk density and porosity showed significant effect on Ks in particular, within the 0–20 cm soil depth (p = 0.04, n = 122).

Among all land uses, runoff coefficient of bare soil was the highest (26%), followed by grassland and agricultural land (Fig. 5a). Among the forest stands, mixed forest had the lowest runoff coefficient followed by shrubs, broadleaved and conifer stands and afforested land (Fig. 5a). There was a large variation within the groups. The values of the coniferous species, ranged from 2.0% for spruce stands to 10.3% for fir stands, the values of broadleaved species ranged from 1.4% for oak stands to 17.3% for mixed deciduous stands.

Considering the annual rainfall partitioning and the climate zones, runoff coefficient was lower under coniferous (3–9%) than broadleaved stands (2–16%) (Fig. 5c). Interception was the most influencing mechanism within coniferous stands, in particular within climate zone Cs (temperate with dry summer). Contrarily, within the broadleaved stands transpiration was the most influencing mechanism in continental climate with no dry season (Df). Regarding soil infiltration, broadleaved showed the highest potential (up to 60%) compared to the coniferous stands (up to 35%).

### Relative effect sizes

**Figure 6:**
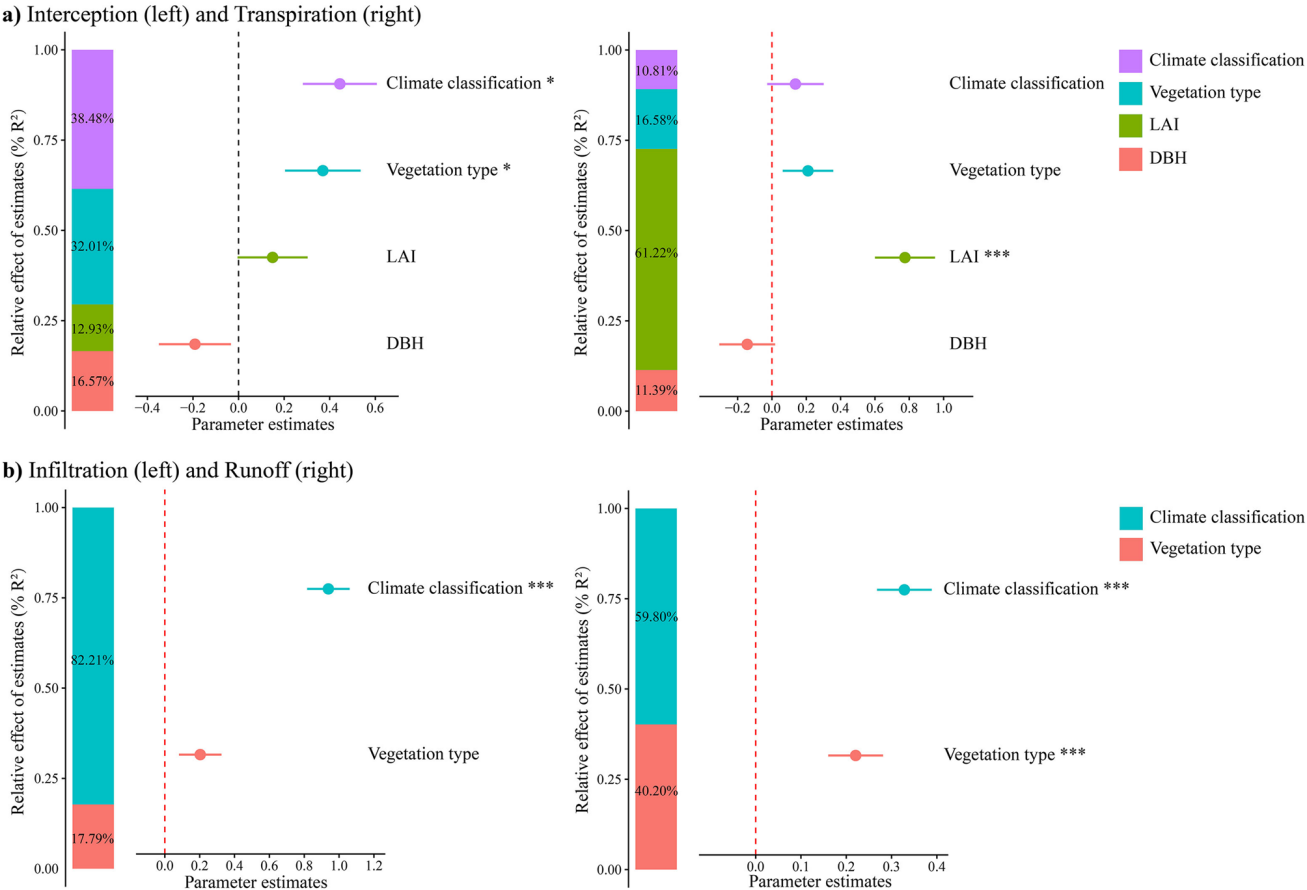
Relative effect of variables: climate, vegetation type, LAI and DBH on (**a**) Interception and transpiration; (**b**) Infiltration and runoff coefficient (*p < 0.05, ***p < 0.001).

To evaluate the relative importance of the influencing factors, we expressed their effect as the percentage of variance they explain, based on the comparison between the absolute values of their standardized regression coefficients and the sum of all standardized regression coefficients. The parameter estimates of the model predictors are shown with their associated standard errors along with the relative importance of each predictor (Fig. 6). This method is similar to a variance partitioning analysis since we previously transformed all factors to z-scores.

Apart from DBH, all other factors positively influenced interception and transpiration. Vegetation types and climate had the biggest influence on interception, whereas for transpiration, LAI was the single most important factor followed by vegetation types, DBH and climate. For infiltration and runoff, climate showed the largest impact. However, vegetation types was significant for runoff coefficient, not for infiltration though.

Climate plays a significant role in overall storm-water management, and therefore, the relative effect size is mostly higher than the vegetation types (Fig. 6). For example, while comparing the runoff coefficient of similar vegetation types (mono-pine stand) at Cw (temperate with dry winter—mean annual precipitation 1370 mm) showed significantly less runoff compared to Cf (Temperate with no dry season—1270 mm) (S-Fig. 1).

Comparative analyses of tree species-specific traits excluding climatic variables are difficult since they directly influence the storm-water partitioning mechanisms. At the same time, data availability for statistical analysis on tree traits is low for identifying particular mechanisms. However, the analyses of specific traits and particular rainfall partitioning mechanisms yielded important insights such as the relative strength of DBH and foliage density on transpiration and interception, crown shape on interception, leaf surface hydrophobicity on interception and rooting depth on soil infiltration (Table 1). For the two biggest components of precipitation partitioning, interception and transpiration, higher foliage density and growth showed the most positive relationship both for conifer and broadleaved species. Every unit of LAI increment revealed an additional 5% rainfall partition through transpiration (3%) and interception (2%). At the same time, site conditions such as tree arrangement displayed counterintuitive results as more open arrangements resulted in higher interception and transpiration rates, while more cluster like arrangements showed an increase in mean soil hydraulic conductivity and a reduction of runoff. Similarly, older trees are mostly important for all the mechanisms of rainfall partitioning except transpiration rate.

**Table 1:** Comparative analyses of site conditions and tree traits on storm-water partitioning.

		Interception	Transpiration	Infiltration	Runoff (reduction)
Traits					
Site conditions					
Tree arrangement		++ (open canopy)	+++ (open canopy)	+ (closed canopy)	+ (closed canopy)
Basic characteristics					
Species origin/habitat		NA	+++ (tropical/riparian)	NA	NA
Age/size (DBH)		+++ (bigger/older)	++ (younger)	+ (older)	+ (older)
Canopy					
Foliage density (LAI)		+++ (high)	+++ (high)	+ (high)	++ (high)
Crown shape		+++ (high vertical LAI)	NA	NA	++ (high vertical LAI)
Canopy height (distance to ground)		NA	NA	NA	++ (shorter)
Branch inclination		+++ (lower)	NA	++ (higher)	NA
Leaf					
Leaf size		++ (small)	NA	NA	+ (small)
Leaf type		++ (needle-leaf)	NA	++ (broadleaf – as litter)	+ (needle-leaf – as litter)
Leaf surface		++ (rough)	NA	NA	+ (rough)
Surface hydrophobicity		+++ (hydrophilic)	NA	NA	+ (hydrophilic)
Stem					
Bark surface texture		++ (rough)	NA	NA	+ (rough)
Wood anatomy		NA	++ (diffuse porous)	NA	NA
Ground cover					
Vegetation cover		+ (high)	+ (high)	+ (high)	+++ (high)
Root type/depth		NA	++ (deep)	+++ (deep)	++ (shallow)
Soil properties					
Total porosity		NA	NA	+++ (high)	+++ (high)
Biotic activity		NA	NA	++ (high)	++ (high)

The ranking of species’ traits in relation to the corresponding rainfall partitioning was from+++(very high effect) to + (low effect), the best categories are in brackets, ‘NA’ refers to both no data and no effect.

Image URL: https://www.anbg.gov.au/cpbr/cd-keys/orchidkey/html/characters/Leaf_surface.htm.

https://cemorowoodcraft.blogspot.com/1991/01/ring-porous-wood.html.

## Discussion

Storm-water control components of rainfall partitioning are interception, transpiration and infiltration, while runoff is the residual from the total precipitation. Climate, vegetation types as well as particular tree traits all influence rainfall partioning separately or conjointly. Concerning the vegetation types, trees with their developed canopy and root systems that increases interception and infiltration^48^, undoubtedly offer the best solution to control storm-water runoff. Oliveira et al.^49^ found that removing matured trees had the potential to increase runoff up to five fold while analyzing the conversion of an undisturbed forest in Mexico to pasture and croplands, and up to 20 fold under bare soil conditions. However, studies also found that the runoff decreases as vegetation mature^50,51^. Conifers tend to have lower antecedent soil water content due to higher interception and transpiration rate resulting in drier soil conditions compared to broadleaf stands, delaying the runoff initiation time and increasing its water holding capacity^52^. All these characteristics suggest that the optimal solution would be the presence of mixed vegetation strata for reducing storm-water runoff. Apparently, only transpiration per unit area decreases; interception, infiltration, increases and runoff decreases with increasing stem density (S-Fig. 2).

### Climate effect

Interception is one of the largest components of rainfall partitioning. Sadeghi et al.^53^ showed an inverse relationship of interception with rainfall intensity and duration; although investigated in non-urban settings, the results could be transferred to urban settings. Subsequently, the highest interception in arid regions and the lowest in the tropical regions. On the other hand, evergreen species with their leaves all year round showed higher interception percentage on annual basis within the climate zones with wet winter such as Cs. Urban trees with comparatively unrestricted crown diameter^54^ and higher vapour pressure deficit^55^ might allure higher retention of rainfall than their rural counterpart. However, interception rate can be less than 10% for storms with depths over 20 mm and rainfall intensity over 7.6 mm h^−1^^56^. Additionally, wind direction and velocity are important since they influence the saturation phase^22^.

Transpiration, another significant component of rainfall partitioning, is more dependent on the soil moisture availability and the rainless higher atmospheric demand situations^57^ such as in the Cf and Df climate zones (with no dry season). Similarly, transpiration from coniferous even within the wet winter climate zones did not show higher amount annually since the most active transpiration takes place during the summer^9^. Although higher amounts of precipitation showed higher transpiration, the trend of soil moisture and tree transpiration is still inconclusive^58^. Moreover, the genetic constituents of different trees with deeper rooting^1^ and stomatal regulations during summer drought^59^ makes it difficult to generalize climatic influence on tree transpiration.

### Vegetation types

The intercepted water mostly comes to the soil and this is soil hydraulic conductivity (Ks), which dictates the amount of available moisture for the rooting system for the subsequent transpiration rate. K_s_ is a complex issue due to the combination of anthropological and geomorphic processes^60^. Woodlands in general have higher Ks compared to agricultural and other land use types. Grassland and shrub land could potentially infiltrate water faster, but had the tendency to saturate faster due to shallow root systems, whereas forest soils infiltrated water slower but had deeper water table. Similarly, conifers such as spruce and pine have shallow rooting system, whilst broadleaf’s such as beech and oaks have deeper rooting system^50,52^. Species with shallow rooting systems did not show much differences at 0–10 cm, but started to slow down further below (S-Fig. 3). This has two big implications, in particular, for cities with higher soil bulk density at the top soil layer, since Ks was found to be negatively related to the bulk density (S-Fig. 4). Any type of vegetation would increase the macro-porosity and reduce the bulk density to at least delay the storm water runoff. On long-terms, broadleaf trees might be better in increasing Ks through deeper rooting system and higher faunal activity through litter fall, which is less acidic than the needle leaves^27^. Evergreen conifers could transpire more compared to deciduous^61^ per annum in particular in temperate regions; however, with low winter precipitation such as New Zealand or Japan, there is no significant difference between annual evapotranspiration from broadleaved and coniferous trees^62^.

### Species characteristics

In general, conifers with higher LAI, leaf area density (LAD), smaller mean distance between leaves as well as less steep leaf angle can intercept more than broadleaved^63^. Among morphological characteristics, DBH and LAI showed the most consistent influence on interception rate. At large, DBH shows a symmetric relationship with growth^64^ and LAI is widely used in quantifying the foliage retention properties^35,65^. Different crown architecture and leaf characteristics also effect the interception rate (Table 1). For instance, an umbrella-shaped canopy tree (*Sophora japonica*) can intercept more^66^ than funnel-shaped canopy tree (*Acer truncatum*) that would direct more water as stem flow instead of dripping off as through-fall^63^. At the same time, trees with higher hydrophobicity (e.g. *Broussonetia papyrifera*) were found to have low interception rate^62^ compared to rigid and rough leaf surface such as *Pistacia chinensis*^63,67^. Similarly, trees with higher LAD such as *Fagus grandifolia* can capture inclined rainfall more efficiently, whereas a greater horizontal and shallower surface canopy i.e. *Liriodendron tulipifera* can capture uninclined droplets more efficiently^68^. The relationship between transpiration and xylem anatomy or sap wood area as well as DBH was inconclusive. The consistent finding was the positive relationship between LAI and transpiration.

Despite mounting evidence of the benefits of trees in storm-water management, there are still limitations to this area of study, particularly in urban settings, where many of the rainfall partitioning data had to be filled in with studies from rural settings. Apart from the limitation of data from rural settings, most studies were confined in North America and Europe, generally in the temperate climate zone (C). In particular, there is a great paucity of transpiration data for tropical (A), arid (B) and polar regions (E). Numerical assessment of urban trees in storm-water management is mostly based on rough estimations^69^ such as in a review by the Centre for Watershed Protection^70^. They found only six studies, three of which used measured data from a single plot, the other three used models. Moreover, tree traits such as leaf area, age or site conditions as e.g. soil^71^ were not considered in most of the studies. Even though standardization was done for better comparability; however, in many cases data were collected at a particular time of the year, which might bias the results. For example, some studies reported interception loss only during the rainy season or transpiration not covering the entire growing season. Additionally, the reviewed studies lack standardized study protocols, methodologies, and addition of site descriptions, relevant morphological and anatomical data especially in tropical, sub-tropical and polar region that could have increased the comparability of studies. Overall, runoff studies in urban settings were limited to the effect of vegetation coverage on runoff, but no empirical study was carried out to compare different functional types of urban vegetation. Results from studies in natural settings should only be applied with great caution in the urban settings as the latter differ in vegetation types, let alone the micro-climatic variations. Moreover, most of the vegetation within urban settings are confined to small growth pits and subjected to soil compaction. Future research incorporating arboricultural (e.g. maintenance practices of individual trees), canopy hydrological processes can help to better calibrate hydrologic models to inform urban planning^69^. In order to avoid ambiguity in terms of morphological characteristics provided in the studies such as foliage density in terms of crown density, one sided leaf area, plant area index, alternative approaches like runoff avoided from volume per unit canopy area should be standardized. Finally, further studies across climate zones with standardized methods of rainfall partitioning mechanisms with different tree traits would allow more accurate and specific treatment to be applied for data normalization for more robust meta-analysis in future.

**Figure 7:**
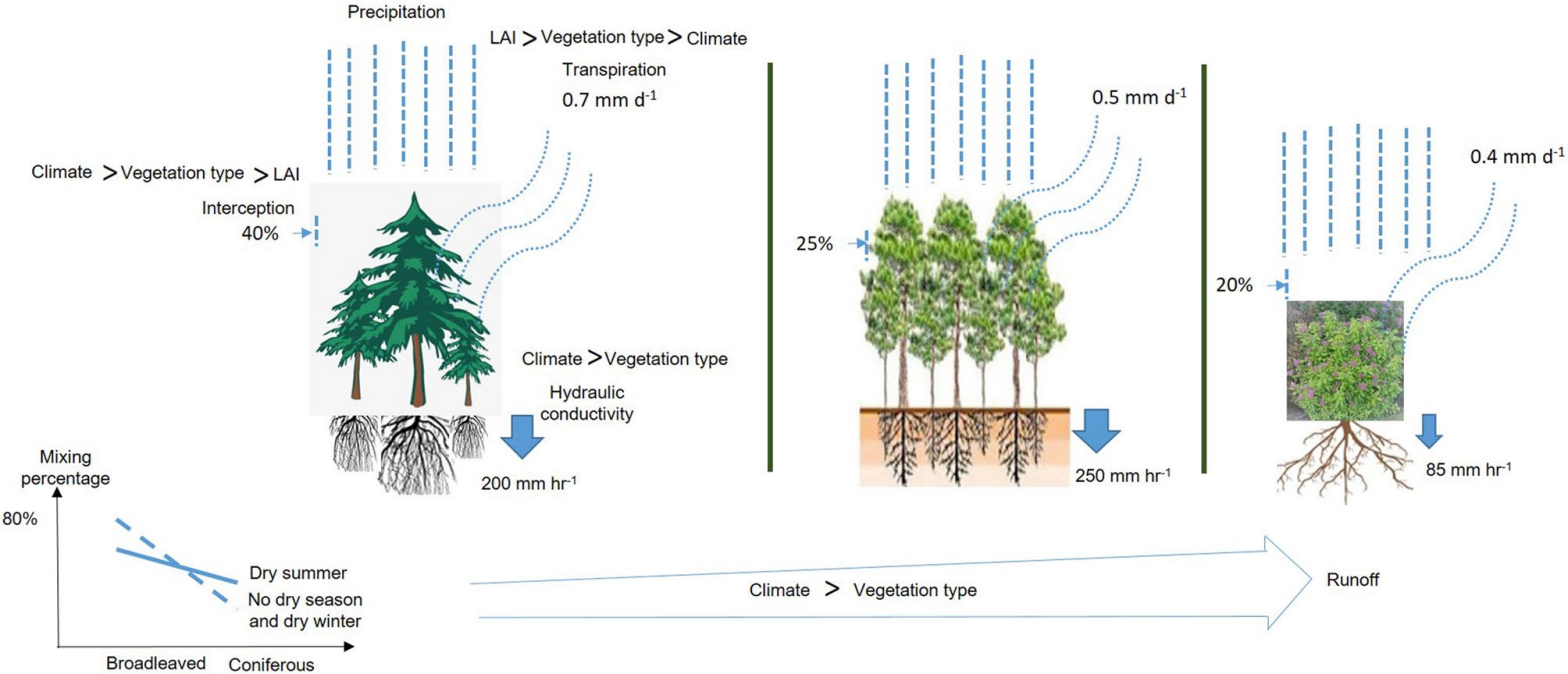
Schematic diagram of major rainfall partitioning pathways by different vegetation types along with the relative effect sizes of variables, consequent runoff coefficient and the proposed species mix for climate zones with distinctive precipitation patterns.

Tree selection for storm-water management should focus on the interception and transpiration partitioning as they are the “first line of defense”, simply because trees in urban watersheds can restore natural hydrologic regimes by higher amounts of interception, transpiration, and infiltration, and consequently, delay of runoff and capture of storm water compared to other types of vegetation^18^. Present selection of species for green infrastructure planning is largely biased by only few species, predominantly broadleaved deciduous trees, at-least in the temperate central Europe^72^. Recent analyses of tree cadasters of 44 central European cities revealed only 5% of conifers within the tree assemblage^73^. The review showed that mixed cultures would be the best solution, but the recipe can vary according to climate zone. In the temperate climate zone with dry summers, a higher proportion of evergreen species (30%) and climate zones with dry winter or no dry season, a higher proportion of deciduous (80%) also with tropical or riparian origin can ultimately lead to lower runoff (Fig. 7). Keeping all other factors constant, the proposed mix can further reduce runoff by around 4%. Within the tropical climate zone, where a foliage-free period is not that pronounced, trees with high LAI, deep and coarse root system can be recommended. In case of arid climates, the focus should be more towards the collection of water for better tree growth. Therefore, species with hydrophobic leaf surfaces, inclined branches (more conical crown) to affluent stem flow as well as deep-rooted species to withstand summer drought would be better.

## Conclusion

With ongoing urbanization and climate change, storm water management has become increasingly important. More attention has been directed towards selection of species or variety of species for planting over hardscaped surfaces such as streets, parking lots or in urban parks having varying impacts on storm water management. Shallow rooted vegetation types including shrubs and grasslands can effectively reduce runoff; however, as soon as the rainfall depth and intensity increases, runoff increases linearly. Thus, big stature vegetation such as trees could potentially intercept more, endure higher stem and through-fall, and improve the soil conditions for increasing water infiltration and transpiration. Among the major rainfall partitioning mechanisms, we have shown that the interception rate is higher in areas with less rainfall but transpiration rate is minimal. Within the vegetation types, conifers showed higher amounts of interception and transpiration water loss at annual scale compared to broadleaved trees, but the soil infiltration rate was higher under the canopies of broadleaved species. Regarding the species traits, canopy density was positively related to both interception and transpiration water loss. Therefore, increasing a mix of conifer and broadleaved species within urban settings will maximise the reduction of storm-water runoff, especially in climate zones with significant winter precipitation or with no distinct dry seasons. However, GI strategies with a narrow focus have practical limits and might not be the most cost-effective option in particular considering the vegetation and land use types along with climate. Therefore, mixed species selection along with shrubs and grasslands to increase the overall functional diversity of greenspaces can facilitate storm-water management. Nevertheless, water attenuation benefits of urban forests are related to a range of ecosystem services, thus, other important regulating (i.e., cooling, air pollution removal, carbon storage); provisioning and cultural services as well as potential disservices such as storm breakage, snow breakage, allergenicity should be taken into account before the final selection. Future comparative research following a standardized protocol can help to understand the broad range of hydrological impacts of diversified urban vegetation across climate regimes.

## Supplementary Information

Supplementary Information.

## Data Availability

Data will be available upon request to the corresponding author.
