# Supplementary material for: A comparative analysis of urban forests for storm-water management
Source: Sci Rep. 2023 Jan 26;13:1451. doi: 10.1038/s41598-023-28629-6 (PMC9879978; doi:10.1038/s41598-023-28629-6)
Supplement: Supplementary file 1 — Supplementary Information. [file 41598_2023_28629_MOESM1_ESM.docx]

**Title: A comparative analysis of urban forests for storm-water management**

# Mohammad A. Rahman^a^*, Yanin Pawijit^a^, Chao Xu^b^, Astrid Moser-Reischl^c^, Hans Pretzsch^c^, Thomas Rötzer^c^, Stephan Pauleit^a^,

^a^Strategic Landscape Planning and Management, School of Life Sciences, Weihenstephan, Technische Universität München, Emil-Ramann-Str. 6, 85354 Freising, Germany

^b^Research Center for Eco-Environmental Engineering, Dongguan University of Technology, Daxue Road 1, 523808 Dongguan, China

^c^Forest Growth and Yield Science, School of Life Sciences, Weihenstephan, Technische Universität München, Hans-Carl-von-Carlowitz-Platz 2, 85354 Freising, Germany

*Corresponding author, Mohammad A RAHMAN, Email: ma.rahman@tum.de, Tel: +49 (0)8161 71 4661

Email address of co-authors:

Yanin PAWIJIT: [yaninpawijit@gmail.com](mailto:yaninpawijit@gmail.com)

Chao XU: chaoxu@dgut.edu.cn

Astrid MOSER-REISCHL: [astrid.moser@tum.de](javascript:linkTo_UnCryptMailto('nbjmup+btusje\/nptfsAmsa\/uvn\/ef');)

Hans PRETZSCH: [hans.pretzsch@tum.de](mailto:hans.pretzsch@tum.de)

Thomas RÖTZER: [thomas.roetzer@tum.de](javascript:linkTo_UnCryptMailto('nbjmup+uipnbt\/spfuafsAmsa\/uvn\/ef');)

Stephan PAULEIT: pauleit@tum.de

S Table 1: Summary of runoff, soil hydraulic conductivity, interception and transpiration data points investigated in this review. Here, c**limate:** according to updated Köppen climate classification, Land use, Mif: Mif: Mixed forest; Af: Afforestation; MiC: Mixed coniferous; MiD: Mixed deciduous; Ag: Agriculture; Sh: Shrub land; Gr: Grassland; Bs: Bare soil; MoF: Mono-Fir stand; MoS: Mono-Spruce stand; MoP: Mono-Pine stand; Ev: Evergreen; MoB: Mono-Beech stand; MoO: Mono-Oak stand; MoBr: Mono-Broadleaved stand; MoE: Mono-Eucalyptus stand; De: Deciduous; TD: Total data points, NA: not applicable, Mo: Modelling, Me: Measured.

1. Run-off

| Study** | Cli-mate | Description of land use /  City* | Land use | | | | | | coniferous | | broadleaved | | | TD | Type of investig-ation | |
| --- | --- | --- | --- | --- | --- | --- | --- | --- | --- | --- | --- | --- | --- | --- | --- | --- |
|  |  |  | **Mif**  **(C / D)** | **Af** | **Ag** | **Sh** | **Gr** | **Bs** | **Mono**  **F / S / P** | **Ev** | **Mono**  **B / O / Br / E** | **De** | **Ev** |  |  |  |
| Hartanto et al., 2003^1^ | A | Tropical rainforest | 4  - | - | - | - | - | - | - / - / - | - | - / - / - / - | - | - | 4 | Me | |
| Patin et al., 2012^2^ | A | Cropland and mixed forest | -  - | - | - | - | 5 | 5 | - / - / - | - | - / - / - / - | - | - | 10 | Me | |
| Cerdà et al., 2017^3^ | B | Secondary forest | - | - | - | - | - | - | - / - / 1 | - | - / 1 / - / - | - | - | 2 | Me | |
| Chirino et al., 2006^4^ | B | Bare soil, grass and shrub land | - | - | - | 1 | 1 | 1 | - / - / 2 | - | - / - / - / - | - | - | 5 | Me | |
| del Campo et al., 2019^5^ | B | Secondary forest | - | - | - | - | - | - | - / - / 1 | - | - / 1 / - / - | - | - | 2 | Me | |
| Descheemaeker et al., 2006^6^ | B | Urban forest | 1  - | - | - | - | 9 | - | - / - / - | - | - / - / - / 1 | - | - | 11 | Me | |
| Quinton et al., 1997^7^ | B | Secondary forest | - | - | - | - | 9 | 2 | - / - / - | - | - / - / - / - | - | - | 11 | Me | |
| Stone et al., 2008^8^ | B | Shrub-grass land | - | - | - | - | 19 | - | - / - / - | - | - / - / - / - | - | - | 19 | Me | |
| Cao et al., 2015^9^ | Cf | Secondary forest | - | - | - | - | - | - | - / - / 5 | - | - / - / - / - | - | - | 5 | Me | |
| Chen et al., 2010^10^ | Cf | Forest stand | 2  - | - | - | - | - | - | - / - / - | - | - / - / - / - | - | - | 2 | Me | |
| El Kateb et al., 2013^11^ | Cf | Maize and tea plantation | -  (- / 5) | - | 5 | - | 3 | - | - / - / 3 | - | - / 3 / - / - | - | - | 14 | Me | |
| Huang et al., 2010^12^ | Cf | Regenerated forest | 1  - | 1 | 1 | - | - | - | - / - / 1 | - | - / - / - / - | - | - | 4 | Me | |
| Hümann et al., 2011^13^ | Cf | Low Alpine afforestation | -  (1 / -) | 2 | 1 | - | - | - | 1 /2 / - | - | 3 / - / - / - | - | - | 11 | Me | |
| Jiang et al., 2019^14^ | Cf | Secondary forest | 1  - | - | - | - | - | - | 1 / - / - | - | - / - / - / - | - | - | 2 | Me | |
| Luo et al., 2020^15^ | Cf | Cropland | 3  - | - | 1 | 1 | - | 1 | - / - / 1 | - | - / - / 3 / - | - | - | 10 | Me | |
| Zheng et al., 2008^16^ | Cf | Afforestation | 1  - | - | - | - | 1 | - | 1 / - | - | - / - / - / 1 | - | - | 5 | Me | |
| Malvar et al., 201317^17^ | Cs | Afforestation | - | - | - | - | - | - | - / - / 1 | - | - / - / - / 6 | - | - | 6 | Me | |
| Mohammad & Adam, 2010^18^ | Cs | Afforestation, natural forest, bare soil | - | - | - | - | 3 | 1 | - / - / - | - | - / - / - / - | - | - | 5 | Me | |
| Nunes et al., 2011^19^ | Cs | Bare soil, grass and shrub land, afforestation | - | - | 1 | 1 | 1 | 1 | - / - / 1 | - | - / 1 / - / - | - | - | 6 | Me | |
| Prats et al., 2012^20^ | Cs | Afforestation | - | - | - | - | - | - | - / - / 2 | - | - / - / - / 2 | - | - | 4 | Me | |
| Thompson et al., 2016^21^ | Cs | Afforestation | - | - | - | - | - | - | - / - / - | - | - / - / - / - | - | - | 2 | Me | |
| Vacca et al., 2000^22^ | Cs | Grass and shrub land, afforestation | - | - | - | - | 3 | - | - / - / - | - | - / - / - / - | - | - | 3 | Me | |
| Gu et al., 2020^23^ | Cw | Crop and shrub land, afforestation | - | - | - | 2 | 2 | - | - / - / - | - | - / - / 2 / - | - | - | 6 | Me | |
| Oliviera et al., 2014^24^ | Cw | Urban woodland | 1  - | - | - | - | - | 1 | - / - / - | - | - / - / - / - | - | - | 2 | Me | |
| Sheridan et al., 2007^25^ | Cw | Eucalyptus forest | - | - | - | - | - | - | - / - / - | - | - / - / - / 2 | - | - | 2 | Me | |
| Sun et al., 2018^26^ | Cw | Afforestation | 3  - | 3 | - | - | - | - | - / - / 1 | - | - / - / - / 2 | - | - | 9 | Me | |
| Wei et al., 2007^27^ | Cw | Cropland | - | - | 1 | 1 | 2 | - | - / - / 1 | - | - / - / - / - | - | - | 5 | Me | |
| Zhang et al., 2006^28^ | Cw | Forest stand | - | - | - | - | - | - | - / - / - | - | - / - / 5 / - | - | - | 5 | Me | |
| Zhou et al., 2002^29^ | Cw | Afforestation | - | - | - | - | - | 1 | - / - / - | - | - / - / 1 / - | - | - | 2 | Me | |
| Holzmann & Sereinig, 1997^30^ | Df | Alpine catchment | 1  - | - | - | - | - | - | - / - / 1 | - | - / - / - / - | - | - | 2 | Me | |
| Minea et al., 2019^31^ | Df | Grassland | - | - | - | - | 1 | 1 | - / - / - | - | - / - / - / - | - | - | 2 | Me | |
| Onuchin et al., 2017^32^ | Df | Secondary forest | - | - | - | - | - | - | 1 / - / - | - | - / - / 2 / - | - | - | 3 | Me | |
| Jost et al., 2012^33^ | Df | Forest stand | - | - | - | - | - | - | - / 1 / - | - | 1 / - / - / - | - | - | 2 | Me | |
| Kim et al., 2014^34^ | Dw | Forest stand | 1  - | - | - | 1 | - | - | - / - / 1 | - | - / 1 / - / - | - | - | 4 | Me | |
| Alaoui et al., 2011^35^ | E | Forest stand | 3  - | - | - | - | 3 | - | - / - / - | - | - / - / - / - | - | - | 6 | Me | |
| Mayerhofer et al., 2017^36^ | E | Grassland | -  (1 / -) | - | - | - | 7 | - | - / - / - | - | - / - / - / - | - | - | 8 | Me | |
| *SUM Runoff* | | | ***22***  ***(2 / 1)*** | ***6*** | ***10*** | ***7*** | ***69*** | ***14*** | ***4 / 3 / 23*** | ***0*** | ***4 / 8 / 13 / 15*** | ***0*** | ***0*** | **201** |  |  |

1. Hydraulic conductivity

| Study** | Cli-mate | Description of land use /  City* | Land use | | | | | | coniferous | | broadleaved | | | *TD* | Type of investig-ation |
| --- | --- | --- | --- | --- | --- | --- | --- | --- | --- | --- | --- | --- | --- | --- | --- |
|  |  |  | ***Mif***  ***(C / D)*** | ***Af*** | **Ag** | **Sh** | **Gr** | **Bs** | **Mono**  **F / S / P** | **Ev** | **Mono**  **B / O / Br / E** | **De** | **Ev** |  |  |
| Johnson-Maynard et al., 2002^37^ | B | Forest stand | - | - | - | 2 | - | - | - / - / 1 | - | - / - / - / - | - | - | 3 | Me |
| Descheemaeker et al., 2006^6^ | B | Urban forest | 1 | - | - | - | 9 | - | - / - / - | - | *- / - / - / 1* | - | - | 11 | Me |
| Chandler et al., 2008^38^ | Cf | Urban forest | - | - | - | - | - | - | - / - / - | - | *- / 1 / - / -* | - | - | 1 | Me |
| Buczko et al., 2006^39^ | Cf | Forest stand | 2 | - | - | - | - | - | - / - / 1 | - | *1 / - / - / -* | - | - | 4 | Me |
| Huemann et al., 2011^13^ | Cf | Low Alpine afforestation | -  (1 / 1)) | 2 | 1 | - | - | - | 1 / 2 / - | - | *3 / - / - / -* | - | - | 11 | Me |
| Neirynck et al., 2000^40^ | Cf | Forest stand | - | - | - | - | - | - | - / - / - | - | *- / 1 / 5 / -* | - | - | 6 | Me |
| Hao et al., 2019^41^ | Cf | Forest stand | 2 | - | - | - | - | - | - / - / - | - | *- / - / - / 1* | - | - | 2 | Me |
| Sheridan et al., 2007^25^ | Cw | Eucalyptus forest | - | - | - | - | - | - | - / - / - | - | *- / - / - / 2* | - | - | 2 | Me |
| Jost et al., 2012^33^ | Db | Forest stand | - | - | - | - | - | - | - / 1 / - | - | *- / - / 1 / -* | - | - | 2 | Me |
| SUM Hydraulic Conductivity | | | ***5***  ***(1 / 1)*** | ***2*** | ***1*** | ***2*** | ***9*** | ***0*** | ***1 / 3 / 2*** | ***0*** | ***4 / 2 / 6 / 3*** | **0** | ***0*** | ***42*** |  |

(c) Interception

| Study** | Cli-mate | Description of land use /  City* | Land use | | | | | | coniferous | | broadleaved | | | *TD* | Type of investig-ation |
| --- | --- | --- | --- | --- | --- | --- | --- | --- | --- | --- | --- | --- | --- | --- | --- |
|  |  |  | ***Mif***  ***(C / D)*** | ***Af*** | **Ag** | **Sh** | **Gr** | **Bs** | **Mono**  **F / S / P** | **Ev** | **Mono**  **B / O / Br / E** | **De** | **Ev** |  |  |
| Nytch et al., 2018^42^ | A | San Juan, Puerto Rico | - | - | - | - | - | - | - / - / - | - | - / - / - / - | 1 | 1 | 2 | Me |
| Park et al., 2008^43^ | A | Afforestation, Soberania, Panama | - | - | - | - | - | - | - / - / - | - | - / - / - / - | 1 | 4 | 5 | Me |
| Guevara-Escobar et al., 2007^44^ | B | Queretaro City, Mexico | - | - | - | - | - | - | - / - / - | - | - / - / - / - | - | 1 | 1 | Mo |
| Sadeghi et al., 2016^45^ | B | Afforestation, Tehran, Iran | - | - | - | - | - | - | - / - / - | 2 | - / - / - / - | 2 | - | 4 | Me |
| Véliz-Chávez et al., 2014^46^ | B | Queretaro City, Mexico | - | - | - | - | - | - | - / - / - | - | - / - / - / - | - | 1 | 1 | Me |
| Fan et al., 2014^47^ | Cf | Afforestation, southeast  Queensland, Australia | - | - | - | 1 | - | - | - / - / - | 1 | - / - / - / - | - | - | 2 | Mo |
| Ford et al., 2011^48^ | Cf | Forest stand, North Carolina, USA | - | - | - | - | - | - | - / - / - | 1 | - / - / - / - | 4 | - | 5 | Me |
| Jiang et al., 2019^14^ | Cf | Secondary forest Sanming City, China | - | - | - | - | - | - | - / - / - | 1 | - / - / - / - | - | 1 | 2 | Me |
| Livesley et al., 2014^49^ | Cf | Melbourne, Australia | - | - | - | - | - | - | - / - / - | - | - / - / - / - | - | 2 | 2 | Mo |
| Ringgaard et al., 2014^50^ | Cf | Forest land, Jutland, Denmark | - | - | - | - | - | - | - / - / - | 3 | - / - / - / - | - | - | 3 | Mo |
| Staelens et al., 2008^51^ | Cf | Forest land, Ghent, Belgium | - | - | - | - | - | - | - / - / - | - | - / - / - / - | 1 | - | 1 | Me |
| Vanstan et al., 2014^52^ | Cf | Forest land, Maryland, USA | - | - | - | - | - | - | - / - / - | - | - / - / - / - | - | 2 | 2 | Me |
| Zabret et al., 2018^53^ | Cf | Ljubljana, Slovenia | - | - | - | - | - | - | - / - / - | 1 | - / - / - / - | 1 | - | 2 | Me |
| Zabret & Šraj, 2019^54^ | Cf | Ljubljana, Slovenia | - | - | - | - | - | - | - / - / - | 1 | - / - / - / - | 1 | - | 2 | Me |
| Asadian et al., 2009^55^ | Cs | British Columbia, Canada | - | - | - | - | - | - | - / - / - | 6 | - / - / - / - | - | - | 6 | Me |
| David et al., 2006^56^ | Cs | Alentejo region, Portugal | - | - | - | - | - | - | - / - / - | - | - / - / - / - | - | 1 | 1 | Me |
| Fathizadeh et al., 2017^57^ | Cs | Forest stand, Southern Zagros, Iran | - | - | - | - | - | - | - / - / - | - | - / - / - / - | 1 | - | 1 | Mo |
| Pereira et al., 2009^58^ | Cs | Herdade da Mitra, Portgual | - | - | - | - | - | - | - / - / - | - | - / - / - / - | - | 2 | 2 | Mo |
| Pypker et al., 2005^59^ | Cs | Forest stand, Gifford Pinchot, USA | - | - | - | - | - | - | - / - / - | 2 | - / - / - / - | - | - | 2 | Mo |
| Xiao et al., 2000^60^ | Cs | California, USA | - | - | - | - | - | - | - / - / - | - | - / - / - / - | 1 | 1 | 2 | Me |
| Xiao & McPherson, 2002^61^ | Cs | Santa Monica, USA | - | - | - | - | - | - | - / - / - | - | - / - / - / - | 3 | 4 | 7 | Mo |
| Xiao & McPherson, 2011^62^ | Cs | California, USA | - | - | - | - | - | - | - / - / - | - | - / - / - / - | 2 | 1 | 3 | Me |
| Ghimire et al., 2012^63^ | Cw | Forest land and afforestation, Nepal | - | - | - | - | - | - | - / - / - | 1 | - / - / - / - | - | 1 | 2 | Mo |
| Negi et al., 1998^64^ | Cw | Forest stand, Central Himalaya, India | - | - | - | - | - | - | - / - / - | 1 | - / - / - / - | 1 | 1 | 3 | Me |
| Buttle et al., 2012^65^ | Df | Forest stand, Ontario, Canada | - | - | - | - | - | - | - / - / - | 2 | - / - / - / - | 1 | - | 3 | Me |
| Price et al., 2003^66^ | Df | Forest stand, Ontario, Canada | - | - | - | - | - | - | - / - / - | - | - / - / - / - | 1 | - | 1 | Mo |
| Liu et al., 2018^67^ | Dw | Forest stand, Beijing, China | - | - | - | 2 | - | - | - / - / - | 3 | - / - / - / - | 5 | - | 10 | Me |
| Yang et al., 2019^68^ | Dw | Seoul, Korea | - | - | - | - | - | - | - / - / - | - | - / - / - / - | 4 | - | 4 | Me |
| *SUM Interception* | | | ***0***  ***(0 / 0)*** | ***0*** | ***0*** | ***3*** | ***0*** | ***0*** | ***0 / 0 / 0*** | ***25*** | ***0 / 0 / 0 / 0*** | ***30*** | ***23*** | ***81*** |  |

(d) Transpiration

| Study** | Cli-mate | Description of land use /  City* | Land use | | | | | | coniferous | | broadleaved | | | *TD* | Type of investig-ation |
| --- | --- | --- | --- | --- | --- | --- | --- | --- | --- | --- | --- | --- | --- | --- | --- |
|  |  |  | ***Mif***  ***(C / D)*** | ***Af*** | **Ag** | **Sh** | **Gr** | **Bs** | **Mono**  **F / S / P** | **Ev** | **Mono**  **B / O / Br / E** | **De** | **Ev** |  |  |
| Ford et al., 2011^48^ | Cf | Forest stands, North Carolina, USA | - | - | - | - | - | - | - / - / - | 1 | - / - / - / - | 4 | - | 5 | Me |
| Jacobs et al., 2015^69^ | Cf | Rotterdam, Netherlands | - | - | - | - | - | - | - / - / - | - | - / - / - / - | 1 | - | 1 | Me |
| Rahman et al., 2019^70^ | Cf | Munich, Germany | - | - | - | - | - | - | - / - / - | - | - / - / - / - | 2 | - | 2 | Me |
| Pataki et al., 2011^71^ | Cs | Los Angeles, USA | - | - | - | - | - | - | - / - / - | 4 | - / - / - / - | 10 | 4 | 18 | Me |
| Raz-Yaseef et al., 2012^72^ | Cs | Forest stand, Israel | - | - | - | - | - | - | - / - / - | 1 | - / - / - / - |  | - | 1 | Me |
| Ghimire et al., 2014^73^ | Cw | Forest stand, Nepal | - | - | - | - | - | - | - / - / - | 1 | - / - / - / - | 1 | - | 2 | Mo |
| Zhu & Zhao, 2013^74^ | Cw | Guangzhou, China | - | - | - | - | - | - | - / - / - | - | - / - / - / - |  | 1 | 1 | Me |
| Matasov et al., 2020^75^ | Df | Moscow, Russia | - | - | - | 1 | - | - | - / - / - | - | - / - / - / - | 3 | - | 4 | Me |
| Peters et al., 2010^76^ | Df | Minnesota, USA | - | - | - | - | - | - | - / - / - | 2 | - / - / - / - | 4 | - | 6 | Me |
| Riikonen et al., 2016^77^ | Df | Helsinki, Finland | - | - | - | - | - | - | - / - / - | - | - / - / - / - | 2 | - | 2 | Me |
| Chen et al., 2011^78^ | Dw | Dalian, China | - | - | - | 1 | - | - | - / - / - | 1 | - / - / - / - | 2 | - | 4 | Me |
| Chen et al., 2012^79^ | Dw | Dalian, China | - | - | - | 1 | - | - | - / - / - | 1 | - / - / - / - | 2 | - | 4 | Me |
| Wang et al., 2011^80^ | Dw | Beijing, China | - | - | - | - | - | - | - / - / - | 2 | - / - / - / - | 4 | - | 6 | Me |
| Wang et al., 2012^81^ | Dw | Beijing, China | - | - | - | - | - | - | - / - / - | - | - / - / - / - | 1 | - | 1 | Me |
| Qiu et al., 2021^82^ | Cw | Shenzhen, China | - | - | - | 3 | - | - | - / - / - | - | - / - / - / - |  | - | - | Me |
| Ji et al., 2016^83^ | Ds | Shrub land, Gansu, China | - | - | - | 3 | - | - | - / - / - | - | - / - / - / - | - | - | - | Me |
| Huang and Zhang, 2016^84^ | B | Shrub land, Zhongwei, China | - | - | - | 2 | - | - | - / - / - | - | - / - / - / - | - | - | - | Me |
| Cavanaugh et al., 2011^85^ | B | Shrub land, Arizona, USA | - | - | - | 1 | - | - | - / - / - | - | - / - / - / - | - | - | - | Me |
| Allen and Grime, 1995^86^ | B | Shrub land, Niger, West Africa. | - | - | - | 1 | - | - | - / - / - | - | - / - / - / - | - | - | - | Me |
| *SUM Transpiration* | | | ***0***  ***(0 / 0)*** | ***0*** | ***0*** | ***13*** | ***0*** | ***0*** | ***0 / 0 / 0*** | ***13*** | ***0 / 0 / 0 / 0*** | ***36*** | ***5*** | ***67*** |  |

*Description of the land use for runoff, soil hydralic conductivity and name of the city for the interception and transpiration urban forest {Patin, 2012 #1318}

** ^1^Hartanto H, Prabhu R, Widayat ASE*, et al.* 2003. Factors affecting runoff and soil erosion: Plot-level soil loss monitoring for assessing sustainability of forest management Forest Ecology and Management **180**: 361-374.

^2^Patin J, Mouche E, Ribolzi O*, et al.* 2012. Analysis of runoff production at the plot scale during a long-term survey of a small agricultural catchment in lao pdr J Hydrol **426-427**: 79-92.

^3^Cerdà A, Lucas Borja ME, Úbeda X*, et al.* 2017. Pinus halepensis m. Versus quercus ilex subsp. Rotundifolia l. Runoff and soil erosion at pedon scale under natural rainfall in eastern spain three decades after a forest fire Forest Ecology and Management **400**: 447-456.

^4^Chirino E, Bonet A, Bellot J*, et al.* 2006. Effects of 30-year-old aleppo pine plantations on runoff, soil erosion, and plant diversity in a semi-arid landscape in south eastern spain CATENA **65**: 19-29.

^5^del Campo AD, González-Sanchis M, Molina AJ*, et al.* 2019. Effectiveness of water-oriented thinning in two semiarid forests: The redistribution of increased net rainfall into soil water, drainage and runoff Forest Ecology and Management **438**: 163-175.

^6^Descheemaeker K, Nyssen J, Poesen J*, et al.* 2006. Runoff on slopes with restoring vegetation: A case study from the tigray highlands, ethiopia J Hydrol **331**: 219-241.

^7^Quinton JN, Edwards GM and Morgan RPC. 1997. The influence of vegetation species and plant properties on runoff and soil erosion: Results from a rainfall simulation study in south east spain Soil Use and Management **13**: 143-148.

^8^Stone JJ, Paige GB, and Hawkins RH. 2008. Rainfall intensity-dependent infiltration rates on rangeland rainfall simulator plots Transactions of the ASABE **51**: 45-53.

^9^Cao L, Liang Y, Wang Y*, et al.* 2015. Runoff and soil loss from pinus massoniana forest in southern china after simulated rainfall CATENA **129**: 1-8.

^10^Chen X, Cheng Q, Chen YD*, et al.* 2010. Simulating the integrated effects of topography and soil properties on runoff generation in hilly forested catchments, south china Hydrol Process **24**: 714-725.

^11^El Kateb H, Zhang H, Zhang P*, et al.* 2013. Soil erosion and surface runoff on different vegetation covers and slope gradients: A field experiment in southern shaanxi province, china CATENA **105**: 1-10.

^12^Huang P and Pretzsch H. 2010. Using terrestrial laser scanner for estimating leaf areas of individual trees in a conifer forest Trees-Struct Funct **24**: 609-619.

^13^Hümann M, Schüler G, Müller C*, et al.* 2011. Identification of runoff processes – the impact of different forest types and soil properties on runoff formation and floods J Hydrol **409**: 637-649.

^14^Jiang M-H, Lin T-C, Shaner P-JL*, et al.* 2019. Understory interception contributed to the convergence of surface runoff between a chinese fir plantation and a secondary broadleaf forest J Hydrol **574**: 862-871.

^15^Luo J, Zhou X, Rubinato M*, et al.* 2020. Impact of multiple vegetation covers on surface runoff and sediment yield in the small basin of nverzhai, hunan province, china Forests **11**: 329.

^16^Zheng H, Chen F, Ouyang Z*, et al.* 2008. Impacts of reforestation approaches on runoff control in the hilly red soil region of southern china J Hydrol **356**: 174-184.

^17^Malvar MC, Martins MAS, Nunes JP*, et al.* 2013. Assessing the role of pre-fire ground preparation operations and soil water repellency in post-fire runoff and inter-rill erosion by repeated rainfall simulation experiments in portuguese eucalypt plantations CATENA **108**: 69-83.

^18^Mohammad AG and Adam MA. 2010. The impact of vegetative cover type on runoff and soil erosion under different land uses CATENA **81**: 97-103.

^19^Nunes AN, de Almeida AC and Coelho COA. 2011. Impacts of land use and cover type on runoff and soil erosion in a marginal area of portugal Applied Geography **31**: 687-699.

^20^Prats SA, MacDonald LH, Monteiro M*, et al.* 2012. Effectiveness of forest residue mulching in reducing post-fire runoff and erosion in a pine and a eucalypt plantation in north-central portugal Geoderma **191**: 115-124.

^21^Thompson A, Davis JD and Oliphant AJ. 2016. Surface runoff and soil erosion under eucalyptus and oak canopy Earth Surface Processes and Landforms **41**: 1018-1026.

^22^Vacca A, Loddo S, Ollesch G*, et al.* 2000. Measurement of runoff and soil erosion in three areas under different land use in sardinia (italy) CATENA **40**: 69-92.

^23^Gu C, Mu X, Gao P*, et al.* 2020. Distinguishing the effects of vegetation restoration on runoff and sediment generation on simulated rainfall on the hillslopes of the loess plateau of china Plant and Soil **447**: 393-412.

^24^Oliveira PTS, Wendland EC, Nearing MA*, et al.* 2014. The water balance components of undisturbed tropical woodlands in the brazilian cerrado Hydrology and Earth System Sciences **19**: 2899-2910.

^25^Sheridan GJ, Lane PNJ and Noske PJ. 2007. Quantification of hillslope runoff and erosion processes before and after wildfire in a wet eucalyptus forest J Hydrol **343**: 12-28.

^26^Sun D, Zhang W, Lin Y*, et al.* 2018. Soil erosion and water retention varies with plantation type and age Forest Ecology and Management **422**: 1-10.

^27^Wei W, Chen L, Fu B*, et al.* 2007. The effect of land uses and rainfall regimes on runoff and soil erosion in the semi-arid loess hilly area, china J Hydrol **335**: 247-258.

^28^Zhang X, Yu X, Wu S*, et al.* 2006. Effect of forest vegetation on runoff and sediment production in sloping lands of loess area Frontiers of Forestry in China **1**: 336-342.

^29^Zhou GY, Morris JD, Yan JH*, et al.* 2002. Hydrological impacts of reafforestation with eucalypts and indigenous species: A case study in southern china Forest Ecology and Management **167**: 209-222.

^30^Holzmann H and Sereinig N. 1997. In situ measurements of hill slope run off components with different types of forest vegetation. In situ measurements of hill slope run off components with different types of forest vegetation. Oxford, UK: International Association of Hydrological Sciences 1997.

^31^Minea G, Ioana-Toroimac G and Moroşanu G. 2019. The dominant runoff processes on grassland versus bare soil hillslopes in a temperate environment - an experimental study Journal of Hydrology and Hydromechanics **67**: 297-304.

^32^Onuchin A, Burenina T and Pavlov I. 2017. Hydrological consequences of timber harvesting in landscape zones of siberia Environments **4**: 51.

^33^Jost G, Schume H, Hager H*, et al.* 2012. A hillslope scale comparison of tree species influence on soil moisture dynamics and runoff processes during intense rainfall J Hydrol **420-421**: 112-124.

^34^Kim JK, Onda Y, Kim MS*, et al.* 2014. Plot-scale study of surface runoff on well-covered forest floors under different canopy species Quaternary International **344**: 75-85.

^35^Alaoui A, Caduff U, Gerke HH*, et al.* 2011. Preferential flow effects on infiltration and runoff in grassland and forest soils Vadose Zone Journal **10**: 367-377.

^36^Mayerhofer C, Meißl G, Klebinder K*, et al.* 2017. Comparison of the results of a small-plot and a large-plot rainfall simulator – effects of land use and land cover on surface runoff in alpine catchments CATENA **156**: 184-196.

^37^Johnson-Maynard JL, Graham RC, Wu L*, et al.* 2002. Modification of soil structural and hydraulic properties after 50 years of imposed chaparral and pine vegetation Geoderma **110**: 227-240.

^38^Chandler KR and Chappell NA. 2008. Influence of individual oak (quercus robur) trees on saturated hydraulic conductivity Forest Ecology and Management **256**: 1222-1229.

^39^Buczko U, Bens O and Hüttl RF. 2006. Water infiltration and hydrophobicity in forest soils of a pine–beech transformation chronosequence J Hydrol **331**: 383-395.

^40^Neirynck J, Mirtcheva S, Sioen G*, et al.* 2000. Impact of tilia platyphyllos scop., fraxinus excelsior l., acer pseudoplatanus l., quercus robur l. And fagus sylvatica l. On earthworm biomass and physico-chemical properties of a loamy topsoil Forest Ecology and Management **133**: 275-286.

^41^Hao M, Zhang J, Meng M*, et al.* 2019. Impacts of changes in vegetation on saturated hydraulic conductivity of soil in subtropical forests Scientific Reports **9**: 8372.

^42^Nytch CJ, Meléndez-Ackerman EJ, Pérez ME*, et al.* 2018. Rainfall interception by six urban trees in san juan, puerto rico Urban Ecosystems **22**: 103-115.

^43^Park A and Cameron JL. 2008. The influence of canopy traits on throughfall and stemflow in five tropical trees growing in a panamanian plantation Forest Ecology and Management **255**: 1915-1925.

^44^Guevara-Escobar A, González-Sosa E, Véliz-Chávez C*, et al.* 2007. Rainfall interception and distribution patterns of gross precipitation around an isolated ficus benjamina tree in an urban area J Hydrol **333**: 532-541.

^45^Sadeghi SMM, Attarod P, Van Stan JT*, et al.* 2016. The importance of considering rainfall partitioning in afforestation initiatives in semiarid climates: A comparison of common planted tree species in tehran, iran Sci Total Environ **568**: 845-855.

^46^Véliz-Chávez C, Mastachi-Loza CA, Gonz¨¢lez-Sosa E*, et al.* 2014. Canopy storage implications on interception loss modeling American Journal of Plant Sciences **Vol.05No.20**: 17.

^47^Fan J, Oestergaard KT, Guyot A*, et al.* 2014. Measuring and modeling rainfall interception losses by a native banksia woodland and an exotic pine plantation in subtropical coastal australia J Hydrol **515**: 156-165.

^48^Ford CR, Hubbard RM and Vose JM. 2011. Quantifying structural and physiological controls on variation in canopy transpiration among planted pine and hardwood species in the southern appalachians Ecohydrology **4**: 183-195.

^49^Livesley SJ, Baudinette B and Glover D. 2014. Rainfall interception and stem flow by eucalypt street trees - the impacts of canopy density and bark type Urban Forestry & Urban Greening **13**: 192-197.

^50^Ringgaard R, Herbst M and Friborg T. 2014. Partitioning forest evapotranspiration: Interception evaporation and the impact of canopy structure, local and regional advection J Hydrol **517**: 677-690.

^51^Staelens J, De Schrijver A, Verheyen K*, et al.* 2008. Rainfall partitioning into throughfall, stemflow, and interception within a single beech (fagus sylvatica l.) canopy: Influence of foliation, rain event characteristics, and meteorology Hydrol Process **22**: 33-45.

^52^Van Stan JT, Van Stan JH and Levia DF. 2014. Meteorological influences on stemflow generation across diameter size classes of two morphologically distinct deciduous species International Journal of Biometeorology **58**: 2059-2069.

^53^Zabret K, Rakovec J and Šraj M. 2018. Influence of meteorological variables on rainfall partitioning for deciduous and coniferous tree species in urban area J Hydrol **558**: 29-41.

^54^Zabret K and Šraj M. 2019. Rainfall interception by urban trees and their impact on potential surface runoff CLEAN – Soil, Air, Water **47**: 1800327.

^55^Asadian Y and Weiler M. 2009. A new approach in measuring rainfall interception by urban trees in coastal british columbia Water Quality Research Journal of Canada **44**: 16-25.

^56^David TS, Gash JHC, Valente F*, et al.* 2006. Rainfall interception by an isolated evergreen oak tree in a mediterranean savannah Hydrol Process **20**: 2713-2726.

^57^Fathizadeh O, Hosseini SM, Zimmermann A*, et al.* 2017. Estimating linkages between forest structural variables and rainfall interception parameters in semi-arid deciduous oak forest stands Sci Total Environ **601-602**: 1824-1837.

^58^Pereira F, Gash JHC, David JS*, et al.* 2009. Evaporation of intercepted rainfall from isolated evergreen oak trees: Do the crowns behave as wet bulbs? Agric For Meteorol **149**: 667-679.

^59^Pypker TG, Bond BJ, Link TE*, et al.* 2005. The importance of canopy structure in controlling the interception loss of rainfall: Examples from a young and an old-growth douglas-fir forest Agric For Meteorol **130**: 113-129.

^60^Xiao Q, McPherson EG, Ustin SL*, et al.* 2000. Winter rainfall interception by two mature open-grown trees in davis, california Hydrol Process **14**: 763-784.

^61^Xiao Q and McPherson EG. 2002. Rainfall interception by santa monica's municipal urban forest Urban Ecosystems **6**: 291-302.

^62^Xiao QF and McPherson EG. 2011. Rainfall interception of three trees in oakland, california Urban Ecosystems **14**: 755-769.

^63^Ghimire CP, Bruijnzeel LA, Lubczynski MW*, et al.* 2012. Rainfall interception by natural and planted forests in the middle mountains of central nepal J Hydrol **475**: 270-280.

^64^Negi GCS, Rikhari HC and Garkoti SC. 1998. The hydrology of three high-altitude forests in central himalaya, india: A reconnaissance study Hydrol Process **12**: 343-350.

^65^Buttle JM and Farnsworth AG. 2012. Measurement and modeling of canopy water partitioning in a reforested landscape: The ganaraska forest, southern ontario, canada J Hydrol **466-467**: 103-114.

^66^Price AG and Carlyle-Moses DE. 2003. Measurement and modelling of growing-season canopy water fluxes in a mature mixed deciduous forest stand, southern ontario, canada Agric For Meteorol **119**: 69-85.

^67^Liu J, Gao G, Wang S*, et al.* 2018. Combined effects of rainfall regime and plot length on runoff and soil loss in the loess plateau of china Earth and Environmental Science Transactions of the Royal Society of Edinburgh **109**: 397-406.

^68^Yang B, Lee DK, Heo HK*, et al.* 2019. The effects of tree characteristics on rainfall interception in urban areas Landscape and Ecological Engineering **15**: 289-296.

^69^Jacobs C, Elbers J, Brolsma R*, et al.* 2015. Assessment of evaporative water loss from dutch cities Building and Environment **83**: 27-38.

^70^Rahman MA, Moser A, Rötzer T*, et al.* 2019. Comparing the transpirational and shading effects of two contrasting urban tree species Urban Ecosystems:

^71^Pataki DE, McCarthy HR, Litvak E*, et al.* 2011. Transpiration of urban forests in the los angeles metropolitan area Ecological Applications **21**: 661-677.

^72^Raz-Yaseef N, Yakir D, Schiller G*, et al.* 2012. Dynamics of evapotranspiration partitioning in a semi-arid forest as affected by temporal rainfall patterns Agric For Meteorol **157**: 77-85.

^73^Ghimire CP, Lubczynski MW, Bruijnzeel LA*, et al.* 2014. Transpiration and canopy conductance of two contrasting forest types in the lesser himalaya of central nepal Agric For Meteorol **197**: 76-90.

^74^Zhu L-w and Zhao P. 2013. Temporal variation in sap-flux-scaled transpiration and cooling effect of a subtropical schima superba plantation in the urban area of guangzhou Journal of Integrative Agriculture **12**: 1350-1356.

^75^Matasov V, Belelli Marchesini L, Yaroslavtsev A*, et al.* 2020. Iot monitoring of urban tree ecosystem services: Possibilities and challenges Forests **11**: 775.

^76^Peters EB, McFadden JP and Montgomery RA. 2010. Biological and environmental controls on tree transpiration in a suburban landscape Journal of Geophysical Research-Biogeosciences **115**:

^77^Riikonen A, Järvi L and Nikinmaa E. 2016. Environmental and crown related factors affecting street tree transpiration in helsinki, finland Urban Ecosystems **19**: 1693-1715.

^78^Chen L, Zhang Z, Li Z*, et al.* 2011. Biophysical control of whole tree transpiration under an urban environment in northern china J Hydrol **402**: 388-400.

^79^Chen L, Zhang Z and Ewers BE. 2012. Urban tree species show the same hydraulic response to vapor pressure deficit across varying tree size and environmental conditions Plos One **7**:

^80^Wang H, Ouyang Z, Chen W*, et al.* 2011. Water, heat, and airborne pollutants effects on transpiration of urban trees Environ Pollut **159**:

^81^Wang H, Wang X, Zhao P*, et al.* 2012. Transpiration rates of urban trees, aesculus chinensis Journal of Environmental Sciences **24**: 1278-1287.

^82^Qiu GY, Wang B, Li T*, et al.* 2021. Estimation of the transpiration of urban shrubs using the modified three-dimensional three-temperature model and infrared remote sensing J Hydrol **594**: 125940.

^83^Ji X, Zhao W, Kang E*, et al.* 2016. Transpiration from three dominant shrub species in a desert-oasis ecotone of arid regions of northwestern china Hydrol Process **30**: 4841-4854.

^84^Huang L and Zhang Z. 2016. Effect of rainfall pulses on plant growth and transpiration of two xerophytic shrubs in a revegetated desert area: Tengger desert, china CATENA **137**: 269-276.

^85^Cavanaugh ML, Kurc SA and Scott RL. 2011. Evapotranspiration partitioning in semiarid shrubland ecosystems: A two-site evaluation of soil moisture control on transpiration Ecohydrology **4**: 671-681.

^86^Allen SJ and Grime VL. 1995. Measurements of transpiration from savannah shrubs using sap flow gauges Agric For Meteorol **75**: 23-41.

S Table 2: Values for plot size normalization (Moreno-de las Heras et al., 2010). Plot names as in the study, here G stands for Gerlach plots (a named method) and MC for ‘microcatchment’.

| **Degraded slopes (slopes 1-3)** | | |  | **Less-degraded slopes (4-5)** | | |
| --- | --- | --- | --- | --- | --- | --- |
| Plot lengths | Runoff coefficient (%) | Reduction of runoff coefficient (%) |  | Plot lengths | Runoff coefficient (%) | Reduction of runoff coefficient (%) |
| G1 (1m) | 79 | 13.92 |  | G1 (1m) | 43 | 55.81 |
| G2 (2m) | 68 | 11.76 |  | G2 (2m) | 19 | 15.79 |
| G3 (3m) | 60 | 3.33 |  | G3 (3m) | 16 | 18.75 |
| MC (15m) | 58 | - |  | MC (15m) | 13 | - |
| MC to G1 |  | 26.58 |  | MC to G1 |  | 69.77 |
| MC to G2 |  | 14.71 |  | MC to G2 |  | 31.58 |

Runoff coefficient: sum of all plot with the same lengths under degraded or less-degraded slopes; Reduction of runoff coefficient: in comparison to the previous shorter length (e.g. from G2 to G1, or G3 to G2)

S Table 3: Values for rainfall intensity normalization (Wu et al., 2018)

| rainfall intensity (mm/hr) | **45** | **60** | **75** | **90** | **105** | **120** |  | **45 to 120** | **45 to 75** | **45 to 105** |
| --- | --- | --- | --- | --- | --- | --- | --- | --- | --- | --- |
| increased rate (of runoff) | - | 1.29 | 1.58 | 1.51 | 1.05 | 1.23 |  | 3.97 | 2.04 | 3.23 |


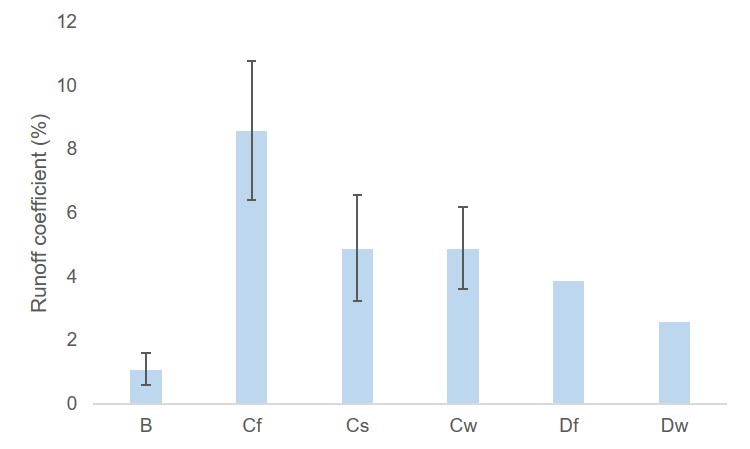


S-Figure 1: Runoff coefficient of Mono-Pine stand (MoP) across climatic zones. Line above bar show standard error


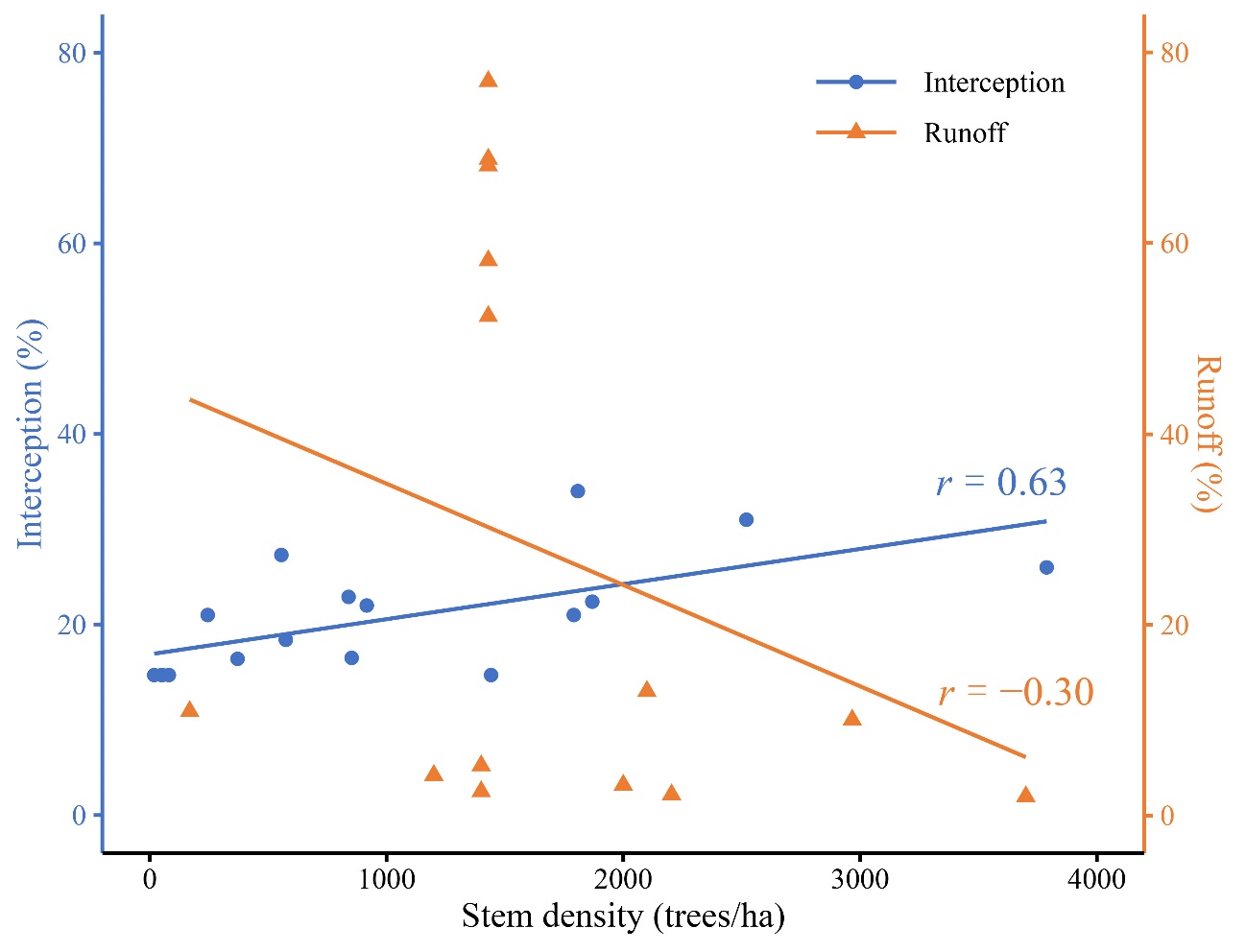


S-Figure 2: Relationship (Pearson correlation coefficient, r) between interception, runoff and stem density.


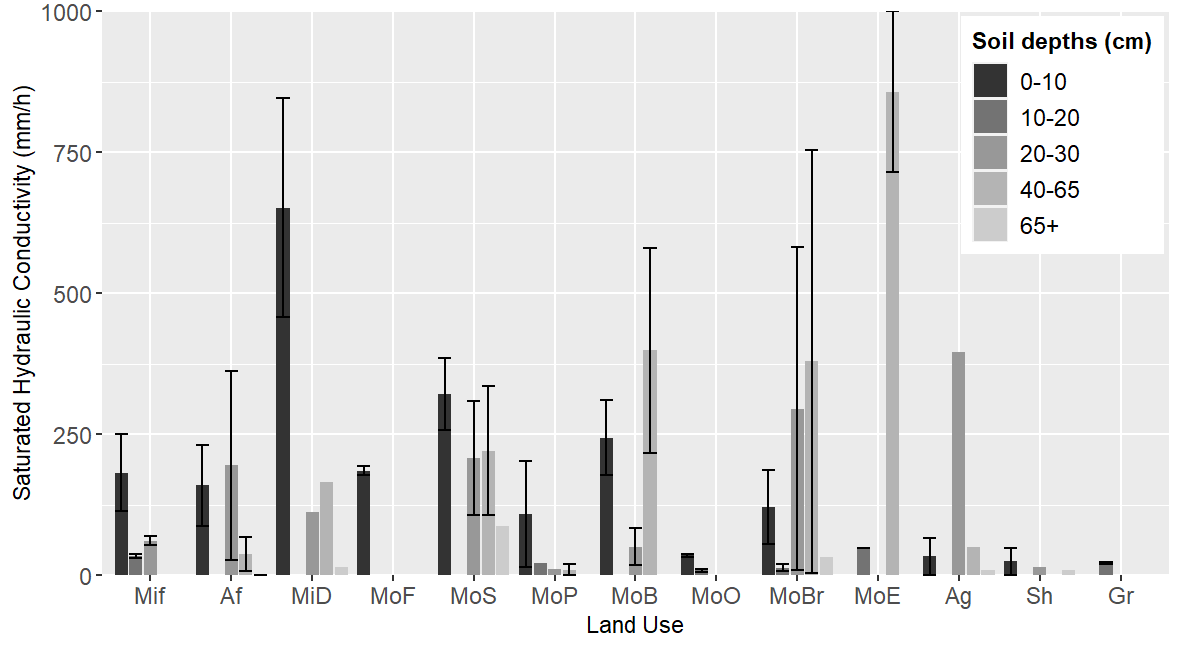


S-Figure 3: Bar chart showing the saturated hydraulic conductivity by land use types at different soil depths with standard error bar. Here, Mif: Mixed forest; Af: Afforestation; MiC: Mixed coniferous; MiD: Mixed deciduous; MoF: Mono-Fir stand; MoS: Mono-Spruce stand; MoP: Mono-Pine stand; MoB: Mono-Beech stand; MoO: Mono-Oak stand; MoBr: Mono-Broadleaved stand; MoE: Mono-Eucalyptus stand; Ag: Agriculture; Sh: Shrubland; Gr: Grassland; Bs: Bare soil.


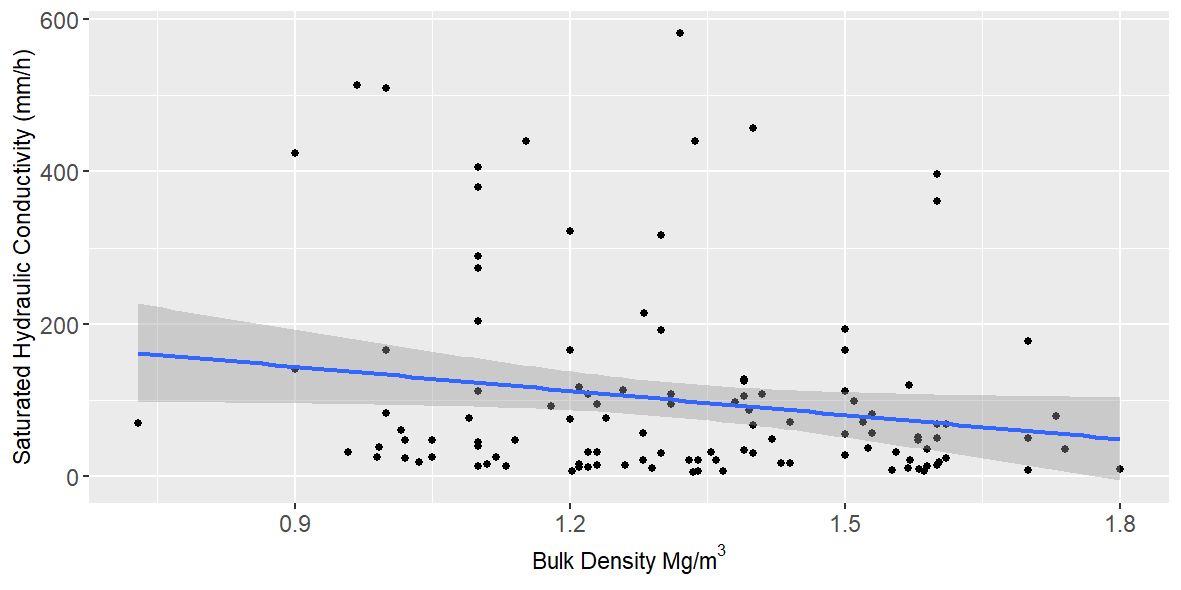


S-Figure 4: Linear regression showing saturated hydraulic conductivity in relation to bulk density
